# Effects and Prognostic Values of Circadian Genes CSNK1E/GNA11/KLF9/THRAP3 in Kidney Renal Clear Cell Carcinoma via a Comprehensive Analysis

**DOI:** 10.3390/bioengineering9070306

**Published:** 2022-07-11

**Authors:** Shujing Li, Xianggang Wang, Qingqing Wang, Kaixin Ding, Xin Chen, Yun Zhao, Yu Gao, Yuanyuan Wang

**Affiliations:** 1 School of Life Science, Bengbu Medical College, Bengbu 233030, China; lishujing@bbmc.edu.cn (S.L.); 20200710010@stu.bbmc.edu.cn (X.W.); 20210710010@stu.bbmc.edu.cn (Q.W.); dingkaixin75@gmail.com (K.D.); chenxin000716@gmail.com (X.C.); zhaoyun0314@gmail.com (Y.Z.); 2 Key Laboratory of Molecular Biophysics of the Ministry of Education, College of Life Science and Technology, Huazhong University of Science and Technology, Wuhan 430074, China; 3 Anhui Province Key Laboratory of Translational Cancer Research, Bengbu Medical College, Bengbu 233030, China

**Keywords:** kidney renal clear cell carcinoma, differentially expressed rhythm genes, immune infiltration, CSNK1E, GNA11, KLF9, THRAP3

## Abstract

Kidney renal clear cell carcinoma (KIRC) is one of the most prevalent and deadly types of renal cancer in adults. Recent research has identified circadian genes as being involved in the development and progression of KIRC by altering their expression. This study aimed to identify circadian genes that are differentially expressed in KIRC and assess their role in KIRC progression. In KIRC, there were 553 differentially expressed rhythm genes (DERGs), with 300 up-regulated and 253 down-regulated DERGs. Functional enrichment analyses showed that DERGs were greatly enriched in the circadian rhythm and immune response pathways. Survival analyses indicated that higher expression levels of CSNK1E were related to shorter overall survival of KIRC patients, whereas lower expression levels of GNA11, KLF9, and THRAP3 were associated with shorter overall survival of KIRC patients. Through cell assay verification, the mRNA level of CSNK1E was significantly up-regulated, whereas the mRNA levels of GNA11, KLF9, and THRAP3 were dramatically down-regulated in KIRC cells, which were consistent with the bioinformatics analysis of KIRC patient samples. Age, grade, stage, TM classification, and CSNK1E expression were all shown to be high-risk variables, whereas GNA11, KLF9, and THRAP3 expression were found to be low-risk factors in univariate Cox analyses. Multivariate Cox analyses showed that CSNK1E and KLF9 were also independently related to overall survival. Immune infiltration analysis indicated that the proportion of immune cells varied greatly between KIRC tissues and normal tissue, whereas CSNK1E, GNA11, KLF9, and THRAP3 expression levels were substantially linked with the infiltration abundance of immune cells and immunological biomarkers. Moreover, interaction networks between CSNK1E/GNA11/KLF9/THRAP3 and immune genes were constructed to explore the stream connections. The findings could help us better understand the molecular mechanisms of KIRC progression, and CSNK1E/GNA11/KLF9/THRAP3 might be used as molecular targets for chronotherapy in KIRC patients in the near future.

## 1. Introduction

Kidney renal clear cell carcinoma (KIRC) is the most common histological subtype of renal cell carcinoma, accounting for 80–90% of all cases, with a high degree of immune infiltration [1,2,3]. A better understanding of the immune system and a discovery that tumors can take advantage of immune checkpoints to promote their survival and growth have resulted in the creation of immune checkpoint inhibitors (ICIs) [4]. Immune checkpoint inhibitors, either alone or in combination, are the new standard of care for treating KIRC, and the rapidly evolving state of systemic therapy for KIRC has prompted clinicians to think about extending immunotherapy to the earlier stages of the disease [5].

The circadian rhythm is critical in maintaining human homeostasis balance. Recent research has discovered that circadian rhythm disorders can lead to a variety of diseases, including endocrine disorders, sleep disorders, metabolic disorders, decreased immune function, neurasthenia, premature aging, and tumors. Circadian rhythm disorders have been linked to an increased risk of cancer in epidemiological studies, and abnormal expression levels of abundant clock genes and circadian reset have also been found in tumor patients [6,7]. In recent bioinformatics research, the expression levels of circadian genes were discovered to be closely associated with the tumor microenvironment of non-small cell lung cancer (NSCLC) [8], and the abnormal expression of circadian genes affected the occurrence and development of glioma by affecting the cell cycle and tumor immune landscape [9]. Our recent study also identified the differentially expressed rhythm genes CSNK1D/CSNK1E/NPAS2 that were closely linked to the occurrence of liver hepatocellular carcinoma (LIHC) and have the potential to be used as diagnostic biomarkers for patients with LIHC [10]. However, bioinformatics studies on differentially expressed rhythm genes in KIRC have only rarely been reported [11,12].

**Figure 1:**
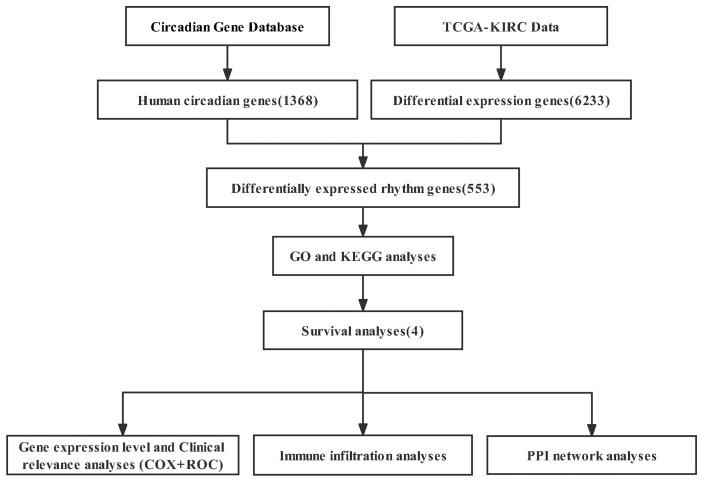
The flow diagram of this study. GO: Gene Ontology; KEGG: Kyoto Encyclopedia of Genes and Genomes.

Here, we used bioinformatics methods to identify the differentially expressed rhythm genes in kidney renal clear cell carcinoma and perform functional enrichment analysis, survival analysis, expression analysis, clinical correlation analysis, immune infiltration analysis, and PPI network analysis (Figure 1). Our study on the relationship between circadian genes and KIRC is expected to provide a theoretical basis for the early diagnosis and clinical prognosis of kidney renal clear cell carcinoma and enable us to better use the circadian clock to assist cancer treatment and promote health.

## 2. Materials and Methods

### 2.1. Data Collection

The Cancer Genome Atlas (TCGA, https://portal.gdc.cancer.gov/, access date: 2 October 2021) was used to obtain gene expression data and clinical information for 539 KIRC tumor samples and 72 normal samples. IMMPORT (https://www.immport.org/, access date: 25 November 2021) provided the list of immunological genes. The data were analyzed using R software (version 4.0.3) with a threshold of *p* < 0.05.

### 2.2. DERGs Identification

We used R software to screen gene expression data and obtained 6233 differentially expressed genes (DEGs) representing differences with |log2-Fold Change (FC)| > 2 and *p*. adjust < 0.05. Using the Venny 2.1 online tool, we intersected 6235 DEGs with 1368 human rhythm genes (http://cgdb.biocuckoo.org/index.php, access date: 31 October 2020) constructed by our team [13] and obtained 300 up-regulated DERGs and 253 down-regulated DERGs. The volcano map of DERGs was drawn by using the ggplot2 package. In the process of differential expression analysis, the Benjamini–Hochberg method was used to correct the significant P-value obtained from the original hypothesis test, and finally, the *p*. adjust value.

### 2.3. Functional Enrichment Analyses

For GO and KEGG pathway analysis, we used the Cluster Profiler program. First, we utilized the limma package to determine the |Log2(Fold Change)| > 2 threshold value of the mRNA expression difference. First, we utilized the limma package to determine the |Log2(Fold Change)| > 2 threshold value of the mRNA expression difference. Subsequently, we used the ggplot2 package in R software for functional enrichment analyses. *p*. adjust < 0.05 was considered statistically significant.

### 2.4. Survival Analyses

The median gene expression was used as the cut-off value for the 539 KIRC samples, which were separated into high and low groups. Using the survminer and survival R packages, we performed the overall survival (OS) analyses of 12 DERGs enriched in the circadian pathway (*p* < 0.05 was considered statistically significant). Then, using the Kaplan–Meier plotter online tool (https://kmplot.com/analysis/, access date: 8 June 2022), the survival curve analyses of the CSNK1E, GNA11, KLF9, and THRAP3 genes were validated (logrank *p* < 0.05 was considered statistically significant). 

### 2.5. Matching Analyses

We matched tumor samples with paracancerous samples from the same patients after evaluating the patient samples (72 pairs in total). Moreover, we extracted the expression levels of CSNK1E, GNA11, KLF9 and THRAP3 genes and drew matching graphs in R software (version 4.0.3).

### 2.6. Immunohistochemical Analyses

The Human Protein Atlas (HPA) provided information on the tissue and cellular distribution of 26,000 human proteins (https://www.proteinatlas.org/, access date: 8 June 2022). We entered the gene name in the search field and clicked ‘SEARCH’. The search results are as follows: first a description of the gene, and then the gene was expressed in tissue, cell, and pathology tumor tissue.

### 2.7. Clinical Correlation Analyses

We compared the expression of CSNK1E, GNA11, KLF9, and THRAP3 in 539 KIRC tumor samples and 72 normal samples using the R software. According to the completeness of clinical information (such as age, sex, and tumor stage), 489 samples were retained for univariate and multivariate COX analyses to evaluate the combined effect of clinical characteristics and genes. The prognostic model was evaluated using area under the curve (AUC) values and the receiver operating characteristic (ROC) curve.

### 2.8. Immune Infiltration Analyses

We applied CIBERSORT to calculate the abundance and correlations of 22 distinct functional immune cell types in KIRC. The relationship between DERGs and immune-infiltrating cells was detected with TIMER2.0, an online analysis website (https://cistrome.shinyapps.io/timer/, access date: 14 October 2021). Moreover, we used TIMER2.0 to analyze the correlation between CSNK1E/GNA11/KLF9/THRAP3 and immune cell gene biomarkers.

### 2.9. Protein–Protein Interaction Network Analyses

We combined the downloaded immune genes with DERGs. The correlation coefficients of DERGs and immune genes were calculated using R software (|cor| > 0.4). CytoScape software was used to map the network between DERGs and differential immune genes.

### 2.10. Cell Culture 

HEK-293T (Procell CL-0005), A-498 (Procell CL-0254) and 786-O (Procell CL-0010) cell lines were kindly provided by Procell Life Science and Technology Co., Ltd. (Wuhan, China), and were correctly identified by STR. RPTEC (FY-22FN1580) cells were kindly provided by Fuyu Biotechnology Co., Ltd. (Wuhan, China), and were correctly identified by STR. HEK-293T and RPTEC cells were cultured in Dulbecco’s modified Eagle’s medium (DMEM, Procell); A-498 and 786-O cells were cultured in Minimum Essential Medium (MEM, iCell) and Roswell Park Memorial Institute (RPMI-1640, Biosharp) separately with 10% fetal bovine serum (FBS, Procell) and 1% penicillin and streptomycin (100 U/mL penicillin and 100 µg/mL streptomycin, Biosharp) in a 37 °C incubator containing 5% CO_2_.

### 2.11. Quantitative Real-Time PCR

Trizol was used to lyse cells and extract total RNA. Quantitative real-time PCR (qRT-PCR) was performed with the TransScript Green One-Step qRT-PCR SuperMix (Trans Gen Biotechnoloy) using the Step One Plus Real-Time PCR System (Life Technologies). The primers of four DERGs were as follows: CSNK1E-fw: ggagatatctacctgggtgcc. CSNK1E-rv: tctcgatgtgcagctggg. GNA11-fw: gaagagcacgttcatcaagc. GNA11-rv: agaggatcttgagcgtctcc. KLF9-fw: cagagtgcatacaggtgaagc. KLF9-rv: tctcacacagcggacagc. THRAP3-fw: gttcttttggggtagtgtctgg. THRAP3-rv: agagcgagatccagacttgg.

### 2.12. Statistical Analysis

All the data were processed and analyzed using the R software (version 4.0.3). We used the Mann–Whitney test and *t*-test to compare the two groups of data. *p* < 0.05 or *p*. adjust < 0.05 was considered statistically significant. 

## 3. Results

### 3.1. DERGs Identification

**Figure 2:**
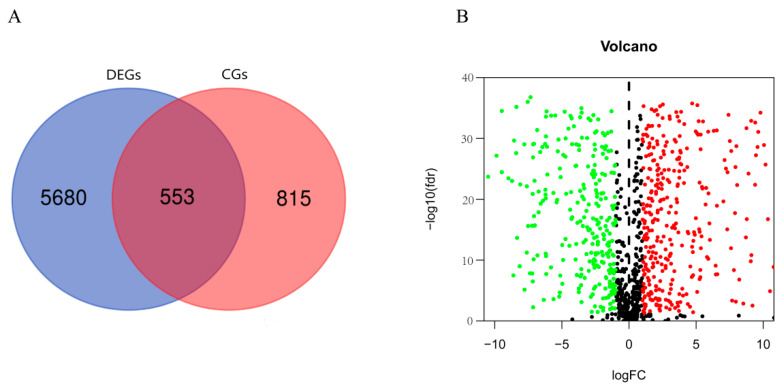
DERGs identification (*p*. adjust < 0.05, |log2FC| > 2). (**A**) Gene cross plot of KIRC and CGDB datasets; (**B**) volcano map of DERGs. The red and green dots represent the up-regulated and down-regulated genes, respectively.

A total of 6233 differentially expressed genes and 1368 circadian genes were noted after analyzing the KIRC-FRPK and CGDB datasets, separately. In total, 553 DERGs were detected in both datasets (Figure 2A, and Table S1). In KIRC tissue samples, 300 genes were up-regulated and 253 genes were down-regulated as compared with normal tissue samples (Figure 2B).

### 3.2. Functional Enrichment Analyses of DERGs

**Figure 3:**
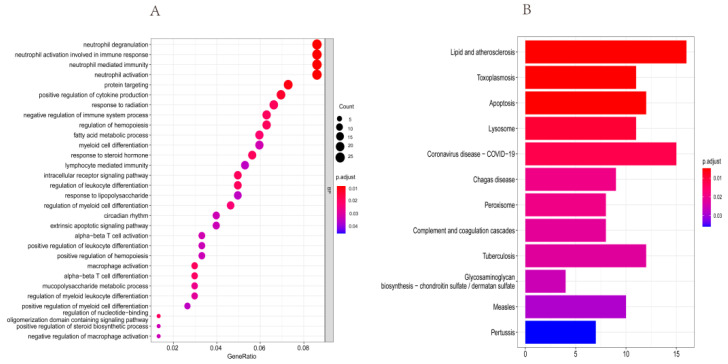
GO and KEGG analyses of DERGs (*p*. adjust < 0.05). (**A**) Biological process enrichment analysis of DERGs; (**B**) KEGG pathway enrichment analysis of DERGs. The redder the color of the dots and columns, the greater the significance. BP: biological process.

Firstly, we made the functional enrichment analysis of 1368 rhythm genes in KIRC, and the results showed that DEGs were significantly enriched in the circadian rhythm and immune signaling pathway (Figure S1). To analyze the function of DERGs, we also performed enrichment analyses of DERGs. It was found that the DERGs were significantly enriched in neutrophil degranulation, neutrophil activation involved in the immune response, neutrophil-mediated immunity, neutrophil activation, and circadian rhythm processes through GO analysis (Figure 3A and Figure S2 and Table S2). The KEGG pathway enrichment analysis indicated that lipid and atherosclerosis, COVID-19, and apoptosis pathways were significantly enriched with *p* < 0.05 (Figure 3B, Table S3).

### 3.3. Survival Analyses of DERGs

**Figure 4:**
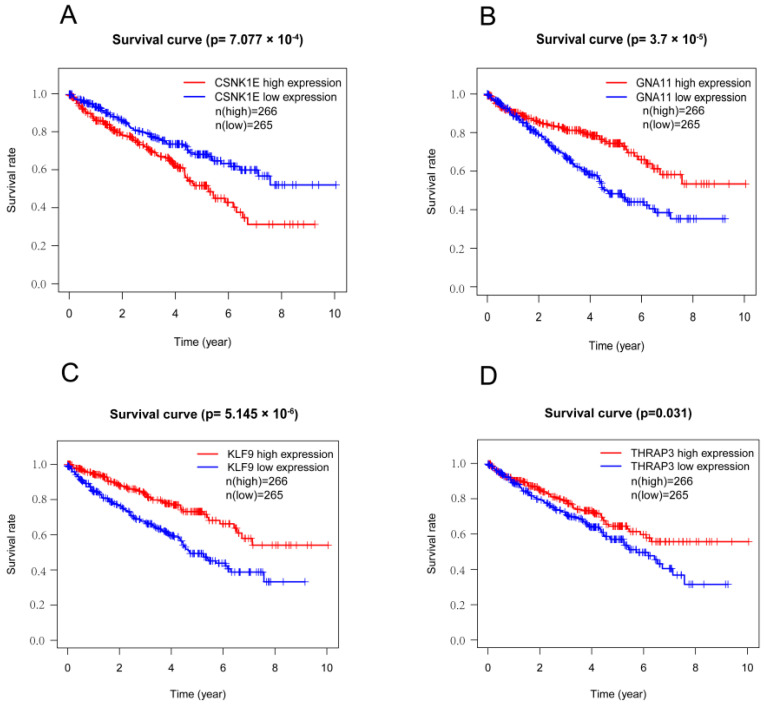
Survival analysis of DERGs genes enriched in the circadian rhythm process. (**A**) CSNK1E; (**B**) GNA11; (**C**) KLF9; and (**D**) THRAP3.

To explore the prognostic values of DERGs, we performed survival analyses of DERGs enriched in the circadian rhythm process. We discovered that the increased expression of CSNK1E was linked with a considerably shorter overall survival in KIRC patients, whereas the decreased expression levels of GNA11, KLF9, and THRAP3 were related to a significantly shorter overall survival in KIRC patients (Figure 4). These findings were further confirmed using the KM plotter online tool (Figure S3). However, HNRNPUL1, TOP1, SREBF1, PER1, SERPINE1, NR1D1, and NR1D2, which were all involved in the circadian rhythm process, did not exhibit a significant relationship with overall survival in KIRC patients (Figure S4).

### 3.4. Expression Level Analyses of DERGs

**Figure 5:**
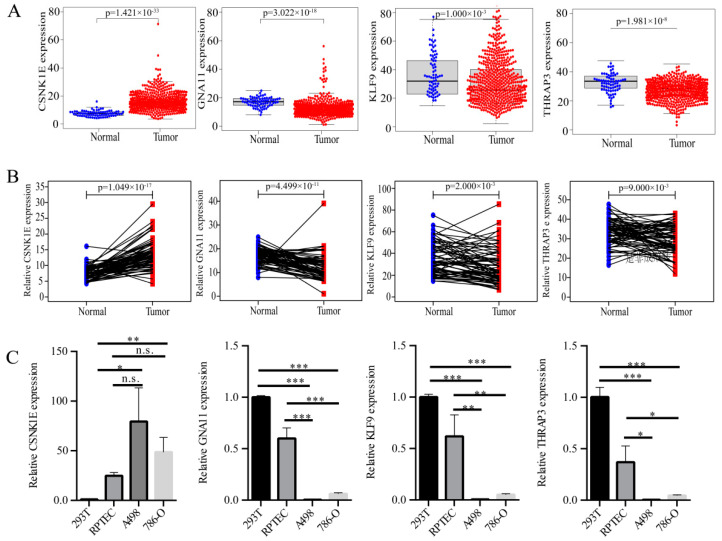
The expression levels of DERGs. (**A**) CSNK1E, GNA11, KLF9, and THRAP3 mRNA levels in 539 KIRC samples and 72 normal samples; (**B**) CSNK1E, GNA11, KLF9, and THRAP3 mRNA levels in KIRC and paracancerous tissues (72 pairs samples); (**C**) CSNK1E, GNA11, KLF9, and THRAP3 mRNA levels in KIRC cells (A498 and 786-O) and control cells (293T and RPTEC) were determined by qRT-PCR (n ≥ 3). The standard deviation (SD) of the mean was represented by the error bars. Student’s *t* test. *** *p* < 0.001, ** *p* < 0.01, * *p* < 0.05.

To investigate the expression levels of CSNK1E/GNA11/KLF9/THRAP3, we analyzed the expression levels of KIRC patients and KIRC cells. Compared with normal tissues, the mRNA levels of CSNK1E were significantly up-regulated, whereas the mRNA levels of GNA11, KLF9, and THRAP3 were strongly down-regulated in 539 KIRC tissues (Figure 5A). We analyzed the patient samples (Table S4) and paired the tumor and paracancerous samples (72 pairs in total), obtaining similar results to before (Figure 5B). We further analyzed the GEO datasets (GSE40435, GSE47032, and GSE46699) and validated our results (Figure S5). Additionally, similar results were obtained in KIRC cell assay, in which the mRNA levels of CSNK1E were strongly increased, whereas the mRNA levels of GNA11, KLF9, and THRAP3 were strongly decreased in A498 and 786-O cells compared with those in 293T and RPTEC cells (Figure 5C). Immunohistochemical staining results of the bioinformatics analysis showed that the protein levels of GNA11 and THRAP3 were significantly decreased in renal cancer tissues, and the protein levels of CSNK1E did not change significantly (Figure S6).

### 3.5. Clinical Relevance Analyses of DERGs

**Figure 6:**
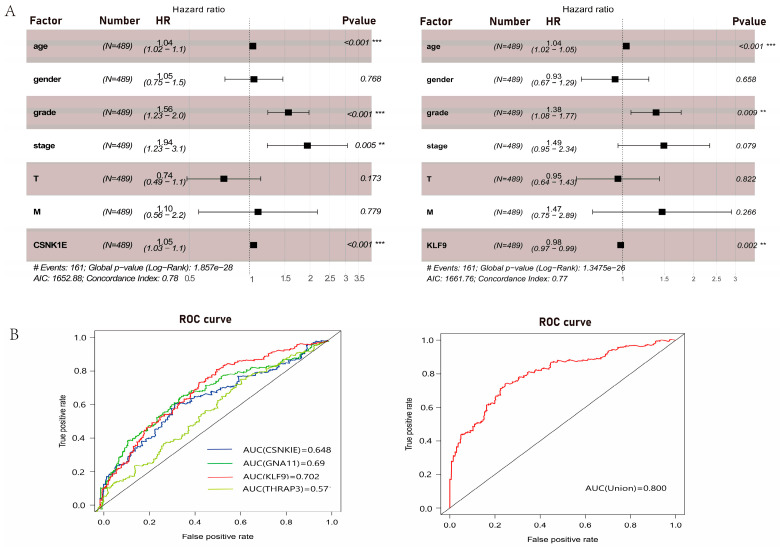
Clinical relevance analyses of DERGs. (**A**) Multivariate Cox analysis of clinical OS in 489 KIRC patients; (**B**) the risk score of ROC curves in KIRC.

To investigate the expression levels and clinical relevance of CSNK1E/GNA11/KLF9/THRAP3, Cox analysis and receiver operating characteristic (ROC) curves for DERGs were derived from clinical data from 489 KIRC patients from the TCGA. Univariate Cox analyses revealed that age, grade, stage, TM classification, CSNK1E expression (HR = 1.03; 95% CI = 1.02–1.05; *p* < 0.001), were high-risk factors, whereas GNA11 expression (HR = 0.92; 95% CI = 0.88–0.96; *p* < 0.001), KLF9 expression (HR = 0.96; 95% CI = 0.95–0.97; *p* < 0.001), and THRAP3 expression (HR = 0.97; 95% CI = 0.95–0.99; *p* < 0.05) were low-risk factors (Table 1, Figure S7, and Table S5). Multivariate Cox analyses showed that CSNK1E (HR = 1.04; 95% CI = 1.02–1.06; *p* < 0.001) and KLF9 (HR = 0.98; 95% CI = 0.96–0.99; *p* < 0.05) were also independently related to overall survival in KIRC patients (Figure 6A). The AUC values of CSNK1E, GNA11, KLF9, and THRAP3 were 0.648, 0.69, 0.702, and 0.57, respectively. Especially when these four genes were analyzed together, the AUC value was 0.8, which had better diagnostic value than the analysis alone (Figure 6B). 

### 3.6. Immune Infiltration Analyses of DERGs

**Figure 7:**
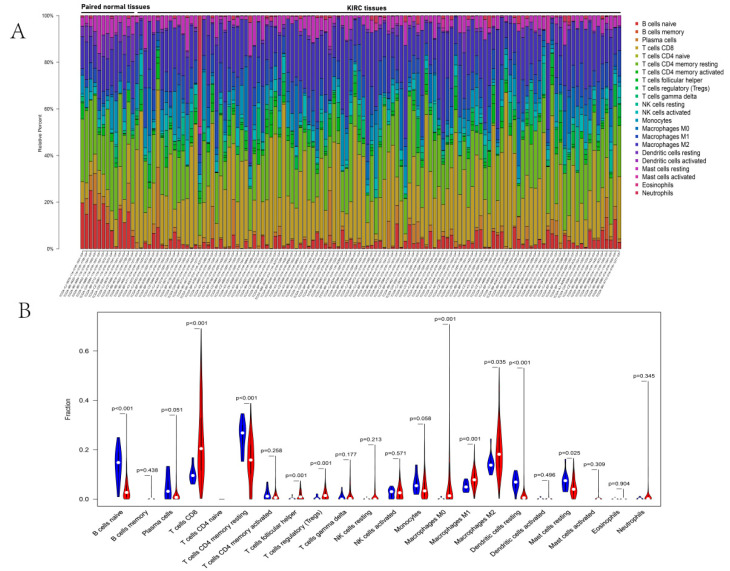
Immune infiltration analyses of DERGs. (**A**) The landscape of immune infiltration between KIRC tissues and paired normal tissues; (**B**) violin plot visualizing differentially infiltrated immune cells. The blue color and red colors represent paired normal tissue and tumor tissue, respectively.

Functional enrichment results revealed a substantial association between DERGs and immune response function. To further confirm the link between DERGs and immune response, we performed immune infiltration analyses in KIRC. The proportion of immune cells in KIRC tissues and normal tissues differed significantly (Figure 7A).

KIRC tissues had more T cells CD8, T cells follicular helper, T cells regulator (Tregs), Macrophages M0, Macrophages M1, and Macrophages M2 than paired normal tissues. However, KIRC tissues had reduced numbers of B cells naive, T cells CD4 memory resting, and dendritic cells activated (Figure 7B). The largest positive correlations were found between the T cell follicular helper and T cell gamma delta (Pearson correlation = 0.74), while T cell CD4 memory resting revealed the strongest negative correlation with T cell CD8 (Pearson correlation = 0.78) and T cell regulatory (Pearson correlation = 0.69). Additionally, dendritic cells resting showed a moderately positive association with T cell CD4 memory resting (Pearson correlation = 0.59), and macrophages M2 showed a moderately negative correlation with plasma cells (Pearson correlation = 0.59) (Figure S8).

### 3.7. Correlation Analyses between DERGs and Immune Infiltration in KIRC

**Figure 8:**
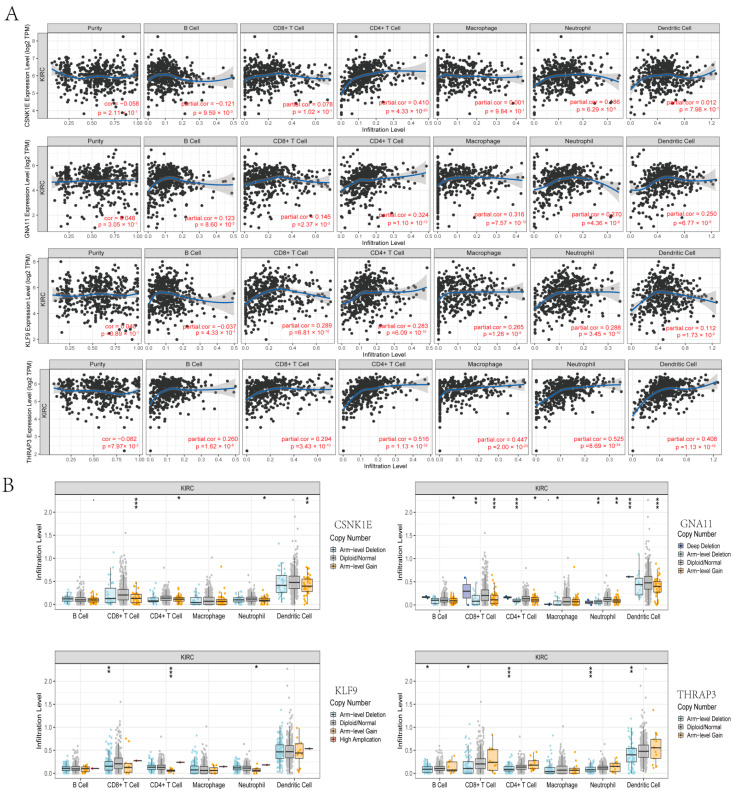
The correlation analyses between DERGs and immune infiltration in KIRC. (**A**) The correlation between the mRNA levels of CSNK1E/GNA11/KLF9/THRAP3 and KIRC immune infiltration; (**B**) the associations of DERGs expression alteration with immune cell infiltration.

To assess whether CSNK1E, GNA11, KLF9, and THRAP3 could be employed as KIRC immunotherapy targets, we examined the connection between CSNK1E/GNA11/KLF9/THRAP3 mRNA levels and KIRC immune infiltration. We found that CSNK1E expression was positively associated with the infiltration abundance of CD4^+^ T cells (r = 0.41, *p* = 4.33 × 10^−^^20^) and neutrophils (r = 0.186, *p* = 6.29 × 10^−^^5^) but was negatively associated with the infiltration abundance of B cells (r = −0.121, *p* = 9.59 × 10^−^^3^) (Figure 8A). GNA11 expression was positively correlated with the abundance of B cells (r = 0.123, *p* = 8.60 × 10^−^^3^), CD8^+^ T cells (r = 0.145, *p* = 2.037 × 10^−^^3^), CD4^+^ T cells (r = 0.324, *p* = 1.10 × 10^−^^12^), macrophages (r = 0.316, *p* = 7.57 × 10^−^^12^), neutrophils (r = 0.27, *p* = 4.36 × 10^−^^9^) and dendritic cells (r = 0.25, *p* = 6.77 × 10^−^^8^) (Figure 8A). Moreover, KLF9 expression was positively correlated with five kinds of immune cell infiltration, including CD8^+^ T cells (r = 0.289, *p =* 6.81 × 10^−^^10^), CD4^+^ T cells (r = 0.283, *p* = 6.09 × 10^−^^10^), macrophages (r = 0.265, *p* = 1.26 × 10^−^^8^), neutrophils (r = 0.288, *p* = 3.45 × 10^−^^10^) and dendritic cells (r = 0.112, *p* = 1.73 × 10^−^^2^) (Figure 8A). B cells (r = 0.26, *p* = 1.62 × 10^−^^8^), CD8^+^ T cells (r = 0.294, *p* = 3.43 × 10^−^^10^), CD4^+^ T cells (r = 0.516, *p* = 1.13 × 10^−^^32^), macrophages (r = 0.447, *p* = 2.00 × 10^−^^32^), neutrophils (r = 0.525, *p* = 8.69 × 10^−^^34^) and dendritic cells (r = 0.408, *p* = 1.12 × 10^−^^19^) were correlated with THRAP3 expression (Figure 8A). Furthermore, we discovered that somatic copy-number alteration (SCNA) of CSNK1E/GNA11/KLF9/THRAP3 altered immunological infiltrates such as CD8^+^ T cells, neutrophils, dendritic cells, macrophages, CD4+ T cells, and B cells (Figure 8B). The findings suggested a link between circadian gene expression and immune infiltration in KIRC.

### 3.8. PPI Network Analyses of DERGs and Immune Genes

**Figure 9:**
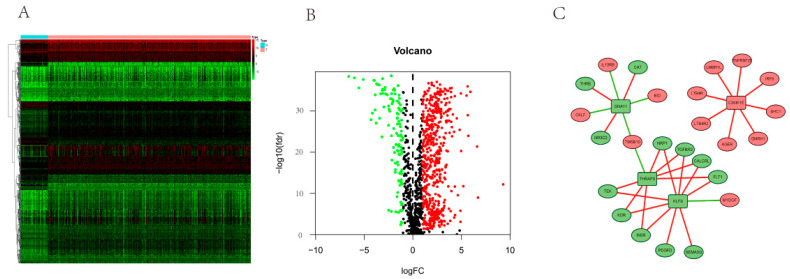
Regulatory networks of DERGs and immune genes. (**A**) Heatmap of immune genes in KIRC; (**B**) volcano plot of differentially expressed immune genes. *p* < 0.05, |log FC| > 2; (**C**) PPI network of DERGs and immune genes. The ellipse and rectangle represented immune genes and DERGs, respectively. The red and green colors represented the up-regulated and down-regulated genes, respectively. The red line represented positive regulation, and the green line represented negative regulation.

To elucidate the regulatory relationships between CSNK1E/GNA11/KLF9/THRAP3 and important immune gene modules, we constructed the PPI network of CSNK1E/GNA11/KLF9/THRAP3 and 101 differentially expressed genes associated with survival (Figure 9 and Figure S9). The interacting immune genes were TMSB10, NR3C2, CKLF, THRB, IL10RB, CAT, BID, LTB4R, LTB4R2, AGER, GNRH1, SHC1, IRF9, TNFRSF25, LMBR1L, TEK, KDR, INSR, PDGFD, SEMA3G, NRP1, TGFBR3, CALCRL, FLT1, and MYDGF.

## 4. Discussion

In epidemiological research, it has been discovered that a disruption in circadian rhythm may increase the risk of cancer, and tumor patients have been found to exhibit an aberrant expression of abundant circadian genes, as well as a reset of their circadian rhythm. In studies examining the role of molecular clocks in human cancers, dysfunctions or polymorphisms in multiple or all of the core circadian genes have been identified. For example, dysregulations or polymorphisms of PER1, PER2, PER3, CLOCK, BMAL1, CRY1, CRY2, and NPAS2 were common in ovarian cancer, prostate cancer, leukemia, gliomas, hepatocellular carcinoma, and colorectal cancer [14,15,16,17,18,19,20,21,22,23,24]. In KIRC patients, bioinformatics methods found that the expression levels of some core clock genes were altered in correlation with the overall survival of patients [3]. However, there are few reports on the relationship between KIRC and other circadian genes in the circadian pathway. In this study, we identified 553 circadian genes whose expression levels were dramatically different in KIRC tissues compared with paired normal tissues. Among them, the expression levels of CSNK1E, GNA11, KLF9, and THRAP3 were clearly related to the survival rate, stage, TM classification, the infiltration abundance of immune cells, and immune biomarkers of KIRC patients.

The protein encoded by CSNK1E was a serine/threonine protein kinase that belonged to the casein kinase I protein family. Members of this family have been linked to cytoplasmic and nuclear activities such as DNA replication and repair. CK1 kinases participated in multiple cellular processes such as apoptosis, differentiation, and cell division [25]. In glioblastoma samples, CSNK1E was expressed at significantly higher levels than in normal tissues [26]. Existing reports have shown that oral squamous cell carcinoma, breast cancer, and colorectal cancer patients with a lower expression of CSNK1E exhibited a considerably longer overall survival rate than patients with higher expression of CSNK1E [26,27,28]. Similarly, we discovered that increased CSNK1E expression was linked to a lower overall survival rate in KIRC patients. Compared with normal tissues, the mRNA level of CSNK1E was significantly up-regulated in KIRC cells and tissues. Univariate Cox analyses showed that age, grade, stage, TM classification and CSNK1E expression were high-risk factors. Immune infiltration analyses showed that CSNK1E expression was positively associated with the infiltration abundance of CD4^+^ T cells and Neutrophils and negatively associated with the infiltration abundance of B cells. CSNK1E positively regulated the expression of differentially expressed immune genes related to survival such as AGER, GNRH1, SHC1, IRF9, TNFRSF25, LMBR1L, LTB4R, and LTB4R2. A recent study reported that AGER, GNRH1, SHC1, IRF9, and LTB4R could be used as prognostic factors of KIRC [29,30,31]. 

The protein encoded by GNA11 was a member of the guanine nucleotide-binding protein (G protein) family, which served as modulators or transducers in a variety of transmembrane signaling systems. The protein encoded by KLF9 was a transcription factor that bound to a GC box element located in the promoter. The encoded protein binding to a single GC box inhibited transcription, while binding to tandem repeating GC Box elements activated transcription. Moreover, KLF9 was a circadian transcription factor in the human epidermis that controlled keratinocyte growth [32]. The protein encoded by THRAP3 had phosphoprotein binding activity, thyroid hormone receptor binding activity and transcription coactivator activity, involving the nuclear-transcribed mRNA catabolic process, positive regulation of the circadian rhythm and regulation of the RNA metabolic process. Recent studies have found that GNA11 mutation was associated with uveal melanoma, the most prevalent primary intraocular malignancy in adults [33]. The expression of KLF9 was low in pancreatic cancer and cutaneous squamous cell carcinoma, and the up-regulation of KLF9 might inhibit the proliferation of pancreatic cancer and cutaneous squamous cell carcinoma [32,34]. However, the link between the three genes and KIRC was unclear. We found that lower expression levels of GNA11/KLF9/THRAP3 were significantly associated with the shorter overall survival of KIRC patients. Compared with normal cells and tissues, the mRNA levels of GNA11/KLF9/THRAP3 were significantly down-regulated in KIRC cells and tissues. Univariate Cox analyses showed that GNA11/KLF9/THRAP3 expression were low-risk factors. GNA11, KLF9, and THRAP3 were positively connected with the expression of differentially expressed immune genes such as TMSB10, NR3C2, CKLF, THRB, IL10RB, CAT, BID, TEK, KDR, INSR, PDGFD, SEMA3G, NRP1, TGFBR3, CALCRL, FLT1, and MYDGF, and positively connected with an abundance of CD8^+^ T cells, CD4^+^ T cells, macrophages, neutrophils, and dendritic cells. Moreover, we analyzed the PD-L1(CD274) expression in KIRC tissues and normal tissues from the TCGA and analyzed the correlation between PD-L1(CD274) expression and circadian gene expression using the TIMER website. Renal tumors are highly immunogenic (they attract immune cells); however, they are rendered dysfunctional through the expression of PD-L1 (and other immune inhibitory molecules) on tumor cells. Therefore, we analyzed the PD-L1(CD274) expression in KIRC tissues and normal tissue from the TCGA and analyzed the correlation between PD-L1(CD274) expression and circadian gene expression using the TIMER website. The results showed that the mRNA levels of PD-L1(CD274) were significantly up-regulated compared with normal tissues. The PD-L1(CD274) expression was positively correlated with the GNA11/KLF9/THRAP3 expression but was not significantly correlated with the CSNK1E expression (Figure S10).

Many anticancer drugs have significant time-of-administration effects, and their biological characteristics, such as absorption, distribution, metabolism, and elimination, are synchronized with people’s circadian rhythms. Once these drugs entered tumor cells, their functions would be regulated by the tumor cells’ circadian cycle, resulting in anticancer drugs with variable anticancer effects and adverse consequences depending on the time of administration. A new mode of chronotherapy has been proposed and combined with clinical treatment of tumor, forming chronotherapy of tumor; that is, radiotherapy and chemotherapy could be carried out at a specific time according to the circadian rhythm, so as to maximize the killing of tumor cells and reduce the toxic and side effects related to radiotherapy and chemotherapy [35,36,37]. Bmal1 could be used as a direct molecular target of paclitaxel, and the combination with paclitaxel at the peak of Bmal1 expression has the best inhibitory effect on TSCC proliferation [38]. In an animal model of brain glioma, when radiotherapy was administered during the peak of Per1 or Per2 expression, the apoptosis rate of glioma cells was greater [39]. Microarray data of human blood transcriptome indicated that the peak of CSNK1E expression was at CT7, and the trough of CSNK1E expression was at CT1, whereas the peak of GNA11 expression was at CT7, and the trough of GNA11 expression was at CT16 [40]. KLF9 acts as a circadian transcription factor in the epidermis, controlling keratinocyte proliferation, and the peak of KLF9 expression was at CT6, while the trough of KLF9 expression was at CT18 [32]. The peak of THRAP3 expression was at ZT20, while the trough of THRAP3 expression was at ZT8 in human blood. We discovered that greater CSNK1E expression levels were related to a shorter overall survival in KIRC patients and that CSNK1E expression levels were higher in KIRC tissues, implying that CSNK1E may promote KIRC proliferation. Therefore, when using chemotherapeutic agents that act on the CSNK1E target, we suggest that scheduling the administration at the low peak of CSNK1E expression level can lead to better therapeutic results. On the contrary, higher GNA11/KLF9/THRAP3 expression levels were associated with longer overall survival of KIRC patients, and GNA11/KLF9/THRAP3 expression levels were lower in KIRC tissues, which implied that GNA11/KLF9/THRAP3 might inhibit the proliferation of KIRC. As a result, when administering chemotherapeutic drugs that operate on GNA11/KLF9/THRAP3 targets, we proposed that administration be timed to coincide with high peaks in GNA11/KLF9/THRAP3 expression levels. Experiments are required to determine the precise expression maxima of these four genes. 

There is increasing evidence that the circadian clock is involved in regulating the tumor microenvironment and immune system. Deng’s research found that the immune checkpoint pathway in macrophages was regulated by circadian genes, which could be potential therapeutic targets for lethal infection [41]. Chen’s research found that microglia infiltration could be regulated by the circadian regulator CLOCK, which might be a novel therapeutic target for GBM [42]. Thomas’s research found that CD4^+^ T cell responses were mediated by an internal cellular circadian oscillator, which could drive rhythmic CD4^+^ T cell immunological responses [43]. Our investigation found that CSNK1E, GNA11, KLF9, and THRAP3 were considerably abundant in immune response pathways and significantly linked with the infiltration abundance of immune cells and immunological biomarkers, suggesting that they could be novel immune treatment targets for KIRC.

Although we have found four circadian genes with prognostic value in KIRC using the TCGA database and validated them using the KM online website, we will conduct more cell experiments to investigate in depth the expression of these four genes and the mechanism of action on KIRC in the future.

## 5. Conclusions

**Figure 10:**
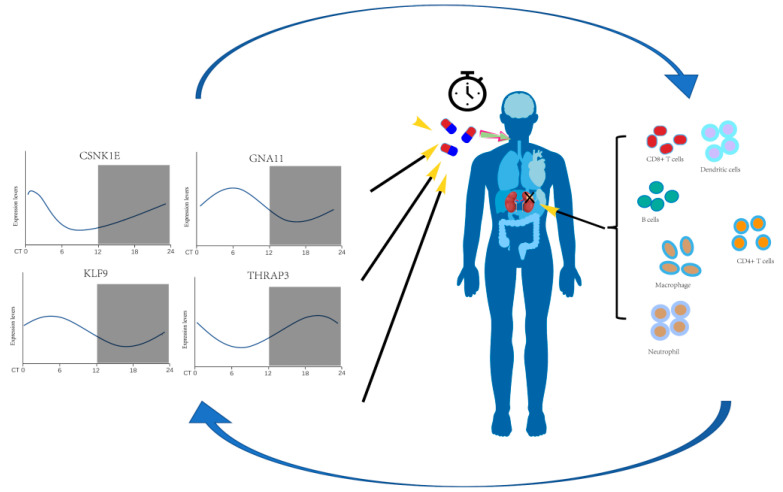
A schematic diagram of the effects and prognostic values of circadian genes CSNK1E/GNA11/KLF9/THRAP3 in kidney renal clear cell carcinoma.

In this study, we identified 553 rhythm genes that were differentially expressed in kidney renal clear cell carcinoma. Among them, the expression levels of CSNK1E, GNA11, KLF9, and THRAP3 were not only significantly correlated with overall survival and other clinical parameters of KIRC patients but also strongly associated with the infiltration abundance of immune cells and immunological biomarkers. In summary, circadian genes, cancer hallmark pathways and immune infiltration are closely related (Figure 10). Our findings could pave the way for the use of personalized chronotherapy and immune checkpoint inhibitors in the treatment of kidney renal clear cell carcinoma.

## Figures and Tables

**Table 1:** Univariate cox analyses of the correlation of CSNK1E/GNA11/KLF9/THRAP3 expression with OS among KIRC patients.

Parameter	Univariate Analysis		
	HR	95% CI	*p*
age	1.03	1.01–1.04	<0.001
grade	2.29	1.85–2.83	<0.001
stage	1.88	1.64–2.16	<0.001
gender	0.93	0.67–1.28	>0.05
T	1.94	1.63–2.29	<0.001
M	4.28	3.10–5.90	<0.001
CSNK1E	1.03	1.02–1.05	<0.001
KLF9	0.96	0.95–0.97	<0.001
THRAP3	0.97	0.95–0.99	<0.05
GNA11	0.92	0.88–0.96	<0.001

CI: confidence interval. HR: hazard ratio; T: topography; M: metastasis.

## Data Availability

Publicly available datasets were analyzed in this study, and these can be found in the database of The Cancer Genome Atlas (https://portal.gdc.cancer.gov) and The Circadian Gene Database (http://cgdb.biocuckoo.org). The authors confirm that the data supporting the findings of this study are available within the article.

## Supplementary Materials

The following supporting information can be downloaded at: https://www.mdpi.com/article/10.3390/bioengineering9070306/s1, Figure S1: GO and KEGG analyses of 1368 circadian genes. (A) GO analysis; (B) KEGG analysis, Figure S2: GO enrichment analysis of DERGs. (A) Cell component enrichment analysis; (B) molecular function enrichment analysis, Figure S3: The survival curves of DERGs by the KM plotter online tool. (A) CSNK1E; (B) GNA11; (C)KLF9; and (D) THRAP3, Figure S4: The correlation between the expression levels of other DERGs and the survival rate of KIRC patients, Figure S5: The expression levels of DERGs in KIRC samples from GEO datasets. (A) CSNK1E; (B) GNA11; (C) KLF9; and (D) THRAP3, Figure S6: Representative images of CSNK1E/ GNA11/ THRAP3 immunohistochemistry in kidney and renal cancer tissues by the human protein atlas online tool (no relevant data found for KLF9 immunohistochemistry), Figure S7: Correlation analysis between DERGs expression and clinical variables. (A) CSNK1E; (B) GNA11; (C) KLF9; and (D) THRAP3, Figure S8: Correlation heatmap depicting correlations between infiltrating immune cells in KIRC, Figure S9: Hazard ratios of 101 differentially expressed immune genes associated with survival, Figure S10: The correlation analysis between PD-L1(CD274) expression and CSNK1E/GNA11/KLF9/THRAP3 expression. (A) The PD-L1(CD274) mRNA level in 539 KIRC samples and 72 normal samples; (B) The correlation between PD-L1(CD274) expression and CSNK1E/GNA11/KLF9/THRAP3 expression with TIMER online tool, Table S1: The 553 DERGs between KIRC samples and normal tissue samples, Table S2: GO analyses, Table S3: The KEGG pathway enrichment analysis, Table S4: The expression levels of DERGs in the normal and KIRC tissues, Table S5: The clinical data of KIRC patients.
