# Supplementary material for: Effects and Prognostic Values of Circadian Genes CSNK1E/GNA11/KLF9/THRAP3 in Kidney Renal Clear Cell Carcinoma via a Comprehensive Analysis
Source: Bioengineering (Basel). 2022 Jul 11;9(7):306. doi: 10.3390/bioengineering9070306 (PMC9311602; doi:10.3390/bioengineering9070306)
Supplement: Supplementary file 1 [file bioengineering-09-00306-s001.zip › Supplementary Table.S1.pdf]

Supplementary Table S1. The 553 DERGs between KIRC samples and normal tissue samples.

ID	conMean	treatMean	logFC	P	P.adjust
ABCA1	3.708622	11.22317	7.514551	2.81E-31	2.73E-30
ABCB1	22.41782	6.986941	-15.4309	5.76E-35	1.14E-33
ABCD1	3.263849	6.661305	3.397456	1.62E-31	1.63E-30
ABCG1	5.00024	12.12079	7.120546	5.52E-28	3.61E-27
ABHD2	17.69889	14.30813	-3.39076	5.30E-14	1.26E-13
ABHD4	17.21864	10.60772	-6.61092	2.29E-29	1.74E-28
ABHD5	6.139994	3.129966	-3.01003	2.44E-35	5.30E-34
ABLIM1	32.70976	16.50872	-16.201	1.34E-34	2.44E-33
ACADSB	31.33015	8.13349	-23.1967	2.12E-35	4.69E-34
ACOX1	16.41348	8.612776	-7.80071	1.03E-30	9.23E-30
ACSL1	73.25728	42.67917	-30.5781	4.62E-18	1.40E-17
ADAMTSL4	0.748161	3.312092	2.563931	1.58E-29	1.23E-28
ADM	12.07184	80.42273	68.3509	4.95E-37	1.90E-35
AGMAT	68.35659	13.51153	-54.8451	5.88E-08	9.89E-08
AGPAT5	8.906813	11.18552	2.278709	0.000262	0.00035
AGTRAP	16.84731	19.92438	3.077071	0.000629	0.00082
AK3	41.77614	15.12057	-26.6556	1.50E-39	1.71E-37
AKAP1	14.59231	10.3092	-4.28311	5.68E-18	1.72E-17
ALKBH2	3.312506	5.454638	2.142132	2.17E-19	7.24E-19
ALOX5AP	3.809421	8.712633	4.903212	2.67E-15	6.88E-15
AMIGO2	5.889988	3.650629	-2.23936	2.42E-17	7.02E-17
ANK3	7.225486	2.98583	-4.23966	9.90E-31	8.90E-30
AOAH	1.455888	6.79414	5.338252	7.55E-30	6.04E-29
AOC3	13.17125	11.05619	-2.11506	0.003837	0.00474
AP1G1	16.40916	11.26206	-5.1471	1.57E-26	9.03E-26
APOL1	25.99648	107.0531	81.05659	5.05E-25	2.56E-24
APOL2	11.78701	30.35295	18.56594	4.11E-32	4.57E-31
APPL1	11.20906	7.7208	-3.48826	8.76E-22	3.45E-21
AQP3	181.3012	38.36542	-142.936	1.11E-34	2.06E-33
AQP9	0.9126	4.128872	3.216272	1.69E-16	4.68E-16
ARAP3	3.677319	7.357245	3.679927	1.07E-15	2.83E-15
ARHGAP26	2.18487	4.63086	2.44599	8.75E-24	3.99E-23
ARHGAP9	0.499732	3.005669	2.505936	1.97E-35	4.41E-34
ARID5B	16.87803	13.13829	-3.73975	1.26E-05	1.84E-05
ARMCX2	18.38109	11.20458	-7.17651	3.30E-22	1.34E-21
ARSA	9.159227	14.38555	5.226323	3.07E-16	8.37E-16
ARSB	6.764135	9.017143	2.253008	4.30E-06	6.45E-06
ASAH1	94.30864	60.2886	-34.02	7.91E-19	2.53E-18
ASL	14.22971	11.38717	-2.84254	8.04E-05	0.000111
ASNS	3.074277	5.472398	2.398121	4.04E-14	9.66E-14
ATP1B1	828.4411	321.4619	-506.979	2.04E-34	3.56E-33

ATP6V0B	44.44569	28.22016	-16.2255	1.16E-26	6.73E-26
B3GNT8	3.554914	1.363082	-2.19183	1.57E-24	7.65E-24
B4GALT5	23.75475	32.83063	9.075883	7.92E-11	1.57E-10
BAG3	31.11988	24.30286	-6.81702	8.05E-12	1.69E-11
BASP1	5.412059	9.958193	4.546134	0.003786	0.00468
BBS2	7.822712	5.277428	-2.54528	3.88E-27	2.35E-26
BCKDHB	10.6194	4.431453	-6.18795	1.10E-35	2.63E-34
BCL2	8.339657	14.15354	5.81388	1.53E-11	3.16E-11
BCL2A1	0.734245	4.224732	3.490487	3.66E-31	3.49E-30
BCL3	8.920746	13.69738	4.776639	1.75E-08	3.04E-08
BCL6	8.165669	12.92138	4.755706	2.24E-12	4.86E-12
BCL7A	7.701682	3.780898	-3.92078	1.03E-30	9.23E-30
BEX2	28.04342	13.75274	-14.2907	5.73E-25	2.89E-24
BIRC3	5.138882	25.90594	20.76706	1.64E-29	1.27E-28
BRCC3	9.277266	4.483319	-4.79395	1.23E-36	4.06E-35
C14orf132	3.878622	1.421125	-2.4575	6.14E-24	2.84E-23
C1orf54	4.916049	10.35624	5.440192	2.10E-23	9.36E-23
C1QA	30.67384	188.6314	157.9576	8.09E-34	1.25E-32
C1QB	23.21478	179.5414	156.3266	2.69E-35	5.80E-34
C1RL	7.904779	12.60623	4.70145	2.87E-13	6.56E-13
C20orf27	6.451381	10.7942	4.342814	8.64E-27	5.08E-26
C2orf40	9.485874	2.44562	-7.04025	1.89E-24	9.11E-24
C5AR1	3.605504	9.359148	5.753644	3.16E-23	1.38E-22
C8orf59	5.510342	9.261309	3.750967	5.81E-25	2.93E-24
CA4	10.62317	4.044866	-6.5783	1.46E-20	5.28E-20
CALCOCO2	14.8272	16.856	2.028796	1.39E-06	2.15E-06
CAMK2N1	67.35548	44.81955	-22.5359	1.65E-19	5.56E-19
CAMKK2	6.808224	9.818812	3.010588	3.22E-24	1.52E-23
CAPRIN1	35.20789	27.59648	-7.61142	2.70E-12	5.83E-12
CASP8	3.051398	5.167046	2.115648	3.53E-27	2.15E-26
CCDC85B	5.374664	14.15027	8.775611	6.19E-18	1.87E-17
CD200	4.497831	14.55638	10.05855	1.41E-30	1.24E-29
CD248	8.726147	29.9107	21.18456	1.82E-23	8.15E-23
CD300LF	0.324951	2.637871	2.31292	1.03E-37	5.17E-36
CD3E	1.760655	11.22003	9.459376	8.19E-29	5.87E-28
CD46	45.51721	31.79655	-13.7207	4.71E-20	1.64E-19
CD5	0.624199	2.728489	2.10429	3.32E-26	1.86E-25
CD83	13.72935	7.514685	-6.21467	4.27E-22	1.72E-21
CD8B	0.386198	4.244911	3.858713	2.84E-29	2.14E-28
CD93	21.28892	45.23347	23.94454	1.38E-14	3.40E-14
CDC42EP2	7.538463	4.82189	-2.71657	9.46E-14	2.22E-13
CDC42SE1	17.75644	20.49778	2.741341	0.000242	0.000324
CDCA7L	1.556092	5.368508	3.812416	1.38E-29	1.08E-28
CDIPT	30.46326	33.65852	3.195268	0.001795	0.00227

CEACAM1	9.381275	4.287133	-5.09414	4.59E-25	2.33E-24
CFLAR	7.285139	10.01466	2.72952	4.84E-10	9.15E-10
CHCHD7	5.325074	3.321312	-2.00376	2.86E-30	2.42E-29
CHMP1A	27.3901	31.35873	3.968631	3.37E-06	5.10E-06
CHPF2	9.113143	14.58862	5.47548	3.97E-23	1.73E-22
CHST11	2.084835	7.156856	5.072021	1.13E-32	1.41E-31
CHST15	5.040471	16.10055	11.06008	3.49E-23	1.52E-22
CHSY1	9.321054	15.34775	6.026693	1.94E-14	4.74E-14
CLEC18B	2.434091	8.341672	5.90758	3.02E-11	6.13E-11
CLEC2D	0.407064	2.439842	2.032777	5.72E-37	2.16E-35
CLYBL	5.769119	3.141868	-2.62725	4.87E-25	2.47E-24
CNPY3	25.61487	41.61806	16.00319	1.61E-23	7.23E-23
CORO1C	12.86158	27.06598	14.2044	1.04E-29	8.27E-29
COX11	9.835101	7.382638	-2.45246	7.62E-23	3.24E-22
CPD	14.74171	12.17356	-2.56815	9.21E-10	1.72E-09
CPNE2	3.969902	7.721785	3.751883	4.39E-26	2.44E-25
CPPED1	11.46699	9.120413	-2.34658	4.80E-17	1.37E-16
CRAT	24.51782	19.37218	-5.14564	1.48E-05	2.14E-05
CREB5	1.370993	3.918881	2.547888	6.59E-25	3.31E-24
CRIP2	26.24171	36.74781	10.5061	8.87E-06	1.31E-05
CSAD	1.59029	4.768674	3.178384	1.24E-13	2.90E-13
CSF2RA	0.940422	3.654181	2.713759	1.91E-30	1.65E-29
CSF2RB	1.354506	3.549223	2.194716	3.25E-23	1.42E-22
CSF3R	0.361273	2.497307	2.136034	4.99E-36	1.32E-34
CSNK1E	7.571308	16.32325	8.751937	1.42E-33	2.08E-32
CSRNP1	25.93427	19.58891	-6.34536	0.00026	0.000347
CST7	2.11586	18.83099	16.71513	2.68E-34	4.54E-33
CTSD	358.2716	492.7713	134.4997	0.000302	0.000402
CTSF	38.62964	30.48287	-8.14677	5.44E-10	1.03E-09
CTSS	10.84666	41.53151	30.68485	2.94E-29	2.21E-28
CUEDC1	7.82613	5.514696	-2.31143	4.10E-14	9.82E-14
CXCL2	2.54818	7.809354	5.261174	8.04E-08	1.34E-07
CYBRD1	24.00065	16.79213	-7.20852	8.99E-09	1.59E-08
CYCS	44.32218	24.96538	-19.3568	1.14E-27	7.25E-27
CYP27A1	19.02721	26.70892	7.681707	0.000344	0.000455
CYP2J2	1.43727	52.21074	50.77346	1.17E-30	1.04E-29
CYP4F2	12.57278	0.401399	-12.1714	4.28E-17	1.22E-16
DBN1	18.77863	11.72747	-7.05115	7.92E-17	2.23E-16
DDIT4	32.32599	145.0283	112.7023	2.24E-31	2.21E-30
DDR1	28.61029	21.50423	-7.10606	3.64E-11	7.36E-11
DDX24	22.57221	15.83585	-6.73636	4.20E-19	1.37E-18
DENND2A	6.320179	3.718882	-2.6013	3.78E-11	7.64E-11
DNAJC16	7.811367	4.471382	-3.33999	3.10E-18	9.51E-18
DNAJC19	13.72759	7.641432	-6.08616	1.65E-25	8.72E-25

DNM1L	12.51149	9.173188	-3.33831	3.77E-23	1.64E-22
DOCK10	1.270712	3.489792	2.21908	1.57E-26	9.03E-26
DOCK4	2.353754	4.745519	2.391765	3.51E-21	1.32E-20
DOK4	10.6495	17.68462	7.035121	6.11E-21	2.26E-20
DTX3L	8.173414	15.39688	7.223463	1.60E-26	9.16E-26
DUSP1	263.0088	508.0493	245.0404	5.04E-12	1.07E-11
DYRK2	2.744663	4.757756	2.013093	1.40E-22	5.82E-22
DYSF	6.811971	19.42915	12.61718	4.99E-28	3.27E-27
EBF1	2.793446	4.920529	2.127083	1.21E-10	2.37E-10
ECE1	20.80825	29.02211	8.213859	9.59E-08	1.59E-07
ECHDC2	5.631123	7.6704	2.039277	0.002043	0.002574
ECHDC3	35.67388	20.62622	-15.0477	4.68E-14	1.12E-13
EGLN1	15.10646	19.67515	4.568692	6.04E-10	1.14E-09
EIF3E	32.67195	46.66813	13.99619	4.18E-17	1.19E-16
EIF4B	67.47185	87.50079	20.02893	2.58E-06	3.93E-06
EML4	8.637393	11.67389	3.036499	1.06E-11	2.22E-11
ENTPD1	3.469673	7.055872	3.586199	3.97E-28	2.64E-27
EPHX2	37.34381	14.85867	-22.4851	1.70E-26	9.71E-26
EPM2AIP1	6.607721	4.544654	-2.06307	2.73E-22	1.12E-21
ERGIC1	15.29676	30.19893	14.90217	8.59E-29	6.15E-28
ESYT2	23.13522	28.21412	5.078894	1.16E-05	1.70E-05
EXOSC7	7.445503	3.940244	-3.50526	7.18E-32	7.68E-31
FAAH2	6.991615	3.940657	-3.05096	6.92E-27	4.10E-26
FABP5	1.39003	5.110483	3.720453	3.53E-28	2.36E-27
FAM102A	20.81446	9.545372	-11.2691	7.37E-30	5.91E-29
FAM104B	7.064486	4.413896	-2.65059	5.76E-36	1.50E-34
FAM120A	30.82743	27.25996	-3.56748	8.57E-07	1.34E-06
FAM49A	1.325616	3.935573	2.609957	5.67E-29	4.13E-28
FAM53B	5.252628	8.224893	2.972265	4.93E-19	1.60E-18
FBLN5	41.05656	12.51782	-28.5387	7.81E-32	8.27E-31
FBXL16	4.415472	13.12623	8.710762	7.45E-15	1.87E-14
FBXO21	21.66203	10.69091	-10.9711	1.42E-31	1.44E-30
FCGBP	6.326709	2.775545	-3.55116	1.67E-09	3.08E-09
FCGR2A	2.956442	10.95811	8.001668	2.18E-30	1.87E-29
FGR	1.999402	5.741112	3.74171	1.98E-28	1.36E-27
FKBP1A	58.67397	91.60547	32.9315	3.15E-24	1.49E-23
FKBP8	75.18431	87.39309	12.20878	0.00078	0.001011
FLOT1	56.90116	68.19959	11.29843	5.86E-08	9.85E-08
FNDC3B	8.756091	13.12637	4.370278	8.36E-16	2.22E-15
FOLR3	3.915703	0.365673	-3.55003	2.43E-37	1.07E-35
FOS	214.3126	134.7357	-79.5769	1.05E-06	1.63E-06
FOSL2	54.94295	38.89941	-16.0435	5.11E-05	7.15E-05
FOXJ1	2.582774	0.288897	-2.29388	3.67E-35	7.59E-34
FOXO1	10.36542	6.433892	-3.93153	5.00E-27	3.00E-26

FSTL1	24.33877	62.91567	38.5769	6.50E-21	2.40E-20
FURIN	31.93264	26.73636	-5.19628	7.23E-05	0.0001
GABARAPL1	84.36011	28.69902	-55.6611	3.00E-37	1.26E-35
GADD45B	72.17843	46.33684	-25.8416	0.001376	0.001755
GALNT14	23.56257	51.17547	27.6129	1.43E-25	7.61E-25
GATA2	9.248066	3.141013	-6.10705	2.79E-30	2.36E-29
GATM	209.6253	49.78761	-159.838	4.09E-08	6.94E-08
GGA2	16.46377	13.53265	-2.93112	8.38E-11	1.66E-10
GIMAP7	13.68629	32.19384	18.50755	4.41E-20	1.54E-19
GK	9.87484	3.711116	-6.16372	1.11E-08	1.95E-08
GNA11	16.93929	12.70962	-4.22967	3.02E-18	9.28E-18
GNAI2	29.26721	46.89073	17.62351	1.22E-22	5.11E-22
GNG2	2.575745	4.720788	2.145044	8.71E-18	2.60E-17
GNS	85.07344	61.508	-23.5654	2.53E-18	7.83E-18
GOLGA4	11.35533	6.674037	-4.68129	3.10E-22	1.26E-21
GOLGA8A	0.516654	3.861289	3.344635	6.98E-17	1.97E-16
GPR27	3.575303	1.011471	-2.56383	1.12E-30	9.98E-30
GPRC5B	24.06027	10.38286	-13.6774	6.82E-32	7.34E-31
GPX7	5.056833	7.382328	2.325495	1.73E-07	2.83E-07
GRASP	4.499951	7.634879	3.134928	2.02E-10	3.91E-10
GRB10	6.63635	19.14037	12.50402	1.74E-27	1.09E-26
GRPEL1	12.44054	10.10828	-2.33226	1.19E-13	2.77E-13
GTF2I	7.181461	4.788265	-2.3932	5.62E-15	1.42E-14
GZMB	0.760946	4.539313	3.778367	3.66E-32	4.09E-31
HAAO	12.07116	16.35591	4.28475	2.80E-05	3.98E-05
HABP4	11.22862	6.49039	-4.73823	1.40E-28	9.76E-28
HACL1	8.441884	4.338954	-4.10293	6.40E-34	1.01E-32
HAPLN3	0.60284	3.17085	2.56801	3.22E-32	3.64E-31
HCK	2.050128	8.59168	6.541553	3.51E-33	4.77E-32
HEPACAM2	11.08079	5.284465	-5.79633	7.08E-36	1.80E-34
HIST1H2BC	2.682779	5.801799	3.11902	3.83E-06	5.77E-06
HIST1H2BD	20.65996	38.85701	18.19704	2.07E-11	4.24E-11
HIST1H3H	0.456987	2.772095	2.315108	5.64E-21	2.09E-20
HIST2H2BE	14.70718	6.366503	-8.34068	9.01E-15	2.24E-14
HKDC1	6.337415	9.111058	2.773643	0.005028	0.006156
HLA-DOA	8.590775	23.07658	14.48581	3.07E-14	7.41E-14
HLX	0.745428	4.359823	3.614395	1.10E-35	2.63E-34
HNRNPA1	83.96315	109.853	25.88989	1.50E-15	3.92E-15
HNRNPU	50.23267	41.61491	-8.61776	1.90E-08	3.29E-08
HP	0.154782	22.07178	21.917	0.000889	0.001149
HPRT1	27.55009	17.05547	-10.4946	3.87E-25	1.97E-24
HPS4	3.249671	5.399452	2.149781	1.23E-24	6.07E-24
HSD17B8	22.78716	15.77633	-7.01083	7.06E-17	1.99E-16
HSPA1A	25.8369	20.02476	-5.81213	8.42E-10	1.57E-09

HSPA1B	34.36926	20.54746	-13.8218	6.12E-19	1.97E-18
HSPA6	0.654533	2.747033	2.0925	2.90E-31	2.81E-30
HSPA9	86.54257	75.37489	-11.1677	5.13E-07	8.15E-07
HTATSF1	33.82728	21.58515	-12.2421	1.10E-22	4.62E-22
IARS	16.28221	13.63785	-2.64435	2.44E-10	4.70E-10
IDH1	20.61761	13.44768	-7.16993	1.98E-07	3.22E-07
IDS	13.68833	18.4847	4.79637	2.86E-14	6.91E-14
IFIT2	10.19798	12.47351	2.275527	0.000212	0.000286
IFIT3	18.05817	24.52856	6.470387	1.29E-08	2.26E-08
IGF2R	18.46043	15.7601	-2.70032	2.98E-05	4.23E-05
IGHD	3.479772	7.861654	4.381882	0.001316	0.001681
IKBIP	3.956008	13.75193	9.795922	1.92E-36	5.86E-35
IL13RA1	27.18502	30.22176	3.036747	0.009904	0.011873
IL1R1	14.21186	10.91095	-3.3009	4.99E-08	8.44E-08
IL6ST	43.848	41.07022	-2.77777	0.027334	0.031599
IMPDH1	4.1779	7.764266	3.586366	8.68E-21	3.17E-20
INHBB	1.785996	25.17631	23.39031	5.40E-38	3.07E-36
IP6K1	14.97593	9.721777	-5.25416	1.50E-31	1.52E-30
IRF1	5.463306	12.014	6.550695	3.27E-20	1.15E-19
IRF2	11.09789	14.1628	3.064906	1.76E-14	4.30E-14
IRF7	3.246385	10.86633	7.619941	1.53E-29	1.19E-28
ITCH	12.31945	10.00235	-2.31711	3.29E-12	7.07E-12
ITGA5	7.631389	29.35315	21.72176	9.55E-32	9.95E-31
ITGA6	27.98663	36.50492	8.518296	0.001083	0.001392
ITGAX	0.539689	5.246233	4.706544	2.83E-38	1.79E-36
ITPK1	14.94286	11.49853	-3.44432	8.71E-17	2.45E-16
ITPKB	14.23015	7.495108	-6.73504	1.33E-22	5.55E-22
KCNE3	5.54053	25.4895	19.94897	5.53E-33	7.28E-32
KCNJ15	35.48048	14.53453	-20.946	2.06E-19	6.89E-19
KCNJ2	1.090568	3.974674	2.884107	1.31E-24	6.42E-24
KIAA0319L	13.15013	10.13126	-3.01887	4.30E-16	1.16E-15
KIAA1147	10.14392	13.57711	3.433188	0.006081	0.0074
KIF16B	6.790822	4.28173	-2.50909	1.49E-25	7.88E-25
LANCL1	24.1355	15.42175	-8.71375	2.03E-24	9.77E-24
LARP4	8.818792	6.394107	-2.42468	7.99E-18	2.39E-17
LAT2	0.871756	3.916974	3.045218	2.92E-35	6.23E-34
LCN2	8.599859	3.208514	-5.39134	8.73E-21	3.19E-20
LDHB	428.8609	146.1951	-282.666	8.55E-39	6.54E-37
LDLR	6.40385	2.576844	-3.82701	9.73E-21	3.55E-20
LGALS9	2.135659	9.548851	7.413193	5.08E-36	1.34E-34
LHFPL2	1.992209	7.892944	5.900734	6.03E-33	7.84E-32
LILRB4	0.450544	3.973963	3.523419	1.48E-36	4.74E-35
LIN7A	4.696997	8.417138	3.720141	0.019413	0.022741
LITAF	63.51005	39.68115	-23.8289	2.94E-19	9.69E-19

LMNB1	1.941583	4.905947	2.964364	2.43E-26	1.38E-25
LMO7	10.97208	4.721159	-6.25092	9.04E-31	8.15E-30
LPCAT1	7.316948	42.07853	34.76158	3.13E-36	8.74E-35
LPCAT2	7.752124	4.107542	-3.64458	1.01E-22	4.26E-22
LRG1	2.489034	5.432122	2.943088	0.000358	0.000474
LRP10	42.5187	25.19086	-17.3278	2.62E-33	3.65E-32
LRP6	7.652517	4.653744	-2.99877	2.06E-20	7.34E-20
LRPPRC	23.78099	16.70167	-7.07932	1.89E-18	5.87E-18
LTB4R	0.519578	3.043058	2.52348	4.49E-38	2.62E-36
LTBR	14.01458	22.91925	8.904667	1.13E-25	6.03E-25
LUC7L3	8.084656	12.76304	4.678381	3.48E-08	5.94E-08
LYNX1	4.68795	2.026408	-2.66154	3.50E-26	1.96E-25
MAGED1	25.38221	22.57075	-2.81146	2.90E-12	6.25E-12
MAGEF1	29.76508	18.59953	-11.1655	2.18E-26	1.24E-25
MAGEH1	23.1117	18.13484	-4.97686	1.45E-13	3.35E-13
MAGOH	12.02667	14.03173	2.005063	1.18E-06	1.84E-06
MAL	329.7434	29.54488	-300.199	1.91E-39	2.02E-37
MAN1A1	58.70056	21.5461	-37.1545	7.66E-38	4.05E-36
MAN1C1	7.81162	1.671384	-6.14024	1.08E-36	3.64E-35
MANSC1	6.956882	4.037623	-2.91926	3.44E-16	9.35E-16
MAP1LC3A	11.74933	9.269289	-2.48004	5.91E-12	1.25E-11
MAP7	14.34124	10.45567	-3.88556	1.63E-08	2.83E-08
MAP9	4.146226	1.723186	-2.42304	4.91E-33	6.52E-32
MAPK1	18.4707	20.73861	2.26791	0.009175	0.01103
MAPKAPK2	38.73471	42.3879	3.653191	0.002895	0.003606
MBOAT2	6.588774	2.229418	-4.35936	8.02E-36	2.01E-34
MCL1	98.67727	103.4544	4.777174	0.032594	0.03744
MDM2	5.168086	9.291632	4.123546	3.24E-26	1.82E-25
METTL5	6.410306	8.543515	2.133209	6.81E-15	1.71E-14
MKNK2	20.10473	23.71629	3.611564	0.000776	0.001006
MME	44.25643	13.73022	-30.5262	5.23E-06	7.81E-06
MPZL1	13.62207	18.93768	5.315613	2.30E-09	4.21E-09
MRPL35	14.7496	9.856476	-4.89312	7.15E-29	5.15E-28
MRTO4	7.670002	11.3127	3.642702	7.57E-24	3.47E-23
MSRB2	14.13076	16.47918	2.348421	0.002407	0.003016
MUC20	14.2137	9.897796	-4.31591	1.64E-22	6.80E-22
MXRA8	9.013715	21.06606	12.05234	3.10E-06	4.70E-06
MYC	12.39718	35.53526	23.13809	3.09E-23	1.35E-22
MYLIP	15.44444	17.75648	2.312034	0.017372	0.020409
MYO1F	0.810585	4.694882	3.884297	1.29E-36	4.24E-35
NAMPT	11.25318	15.0026	3.749424	2.71E-05	3.86E-05
NAPA	14.99125	12.30935	-2.6819	4.54E-18	1.38E-17
NAT8B	19.28531	12.13892	-7.14638	0.004582	0.005624
NCF4	1.7352	7.074388	5.339188	2.00E-32	2.36E-31

NDRG2	33.77022	13.21895	-20.5513	7.42E-32	7.90E-31
NDUFAF2	10.25813	12.29903	2.040906	0.001492	0.001899
NDUFS5	164.2938	212.0821	47.78828	0.000157	0.000212
NFAM1	0.668158	2.910535	2.242377	5.15E-34	8.26E-33
NFE2L3	3.722489	9.644068	5.921579	2.68E-22	1.10E-21
NFKB2	7.662133	17.83473	10.1726	3.30E-27	2.01E-26
NOP58	10.08866	12.58093	2.492267	3.42E-09	6.18E-09
NPM1	90.75706	172.8352	82.0781	9.76E-29	6.92E-28
NR1D1	9.368591	15.82547	6.456881	4.36E-10	8.27E-10
NR3C2	11.14804	2.762161	-8.38588	1.39E-37	6.69E-36
NSA2	14.61197	21.93084	7.318866	3.28E-16	8.92E-16
NSFL1C	11.1629	13.17457	2.011677	3.47E-12	7.46E-12
NT5C2	7.268386	4.148441	-3.11994	8.28E-32	8.71E-31
NT5DC1	8.556908	6.171785	-2.38512	2.57E-22	1.05E-21
NT5E	6.127113	8.40577	2.278657	0.000192	0.000259
NUCB1	100.3053	109.5047	9.199331	0.002418	0.00303
NUCB2	5.382713	8.744811	3.362098	3.74E-26	2.09E-25
NUCKS1	63.85503	51.05014	-12.8049	2.33E-12	5.05E-12
NYNRIN	6.33999	3.384603	-2.95539	4.12E-16	1.12E-15
OASL	1.068943	3.167357	2.098414	7.52E-24	3.45E-23
OAZ2	12.70542	18.41271	5.70729	2.04E-20	7.30E-20
OCIAD2	32.58258	46.48493	13.90235	1.12E-07	1.86E-07
OFD1	4.667643	7.476882	2.809238	7.87E-17	2.22E-16
OPLAH	7.592036	5.265864	-2.32617	1.18E-13	2.75E-13
ORM1	0.132599	3.962467	3.829868	0.000614	0.000801
ORM2	0.076579	2.804391	2.727812	7.94E-13	1.77E-12
OSTM1	7.772156	10.51506	2.742907	2.73E-11	5.55E-11
P2RY13	1.040583	3.482637	2.442055	4.61E-23	1.99E-22
PABPC1L	0.912772	4.341194	3.428421	2.18E-16	6.00E-16
PACSIN2	30.64611	24.44486	-6.20126	4.08E-12	8.72E-12
PADI2	14.23609	2.525785	-11.7103	5.58E-29	4.07E-28
PAIP2B	4.849584	2.760319	-2.08927	2.64E-21	1.00E-20
PAQR8	7.767149	3.037197	-4.72995	1.40E-14	3.45E-14
PARL	19.22027	24.4857	5.265435	8.60E-16	2.28E-15
PCYOX1	53.52801	30.18457	-23.3434	1.14E-31	1.17E-30
PDK1	1.32196	5.05825	3.73629	7.51E-35	1.45E-33
PDLIM7	4.96367	9.946593	4.982923	4.24E-18	1.29E-17
PEBP1	663.8088	285.8429	-377.966	9.00E-33	1.14E-31
PELI2	5.099336	2.226575	-2.87276	5.37E-35	1.08E-33
PEX7	9.371039	4.857748	-4.51329	1.67E-33	2.42E-32
PFKFB3	101.8757	41.65926	-60.2165	0.039439	0.045011
PFKFB4	0.961429	5.080214	4.118785	2.61E-34	4.44E-33
PHC2	29.97042	37.78913	7.818712	2.43E-09	4.43E-09
PHGDH	15.55599	6.07692	-9.47907	6.45E-26	3.53E-25

PICALM	56.72044	53.17849	-3.54194	0.000618	0.000806
PILRA	2.016723	6.333979	4.317255	5.36E-30	4.38E-29
PITPNA	24.37523	20.75406	-3.62117	2.76E-08	4.73E-08
PJA1	12.47791	7.172888	-5.30502	1.70E-29	1.31E-28
PLAGL1	1.83617	4.418176	2.582006	2.69E-21	1.02E-20
PLAUR	1.890215	5.432327	3.542113	2.41E-21	9.23E-21
PLCB2	0.808865	3.622451	2.813585	4.12E-31	3.90E-30
PLEK	2.499327	12.07706	9.577732	3.13E-30	2.63E-29
PLEKHB1	6.765445	1.277988	-5.48746	6.65E-36	1.70E-34
PLEKHG3	7.36281	3.651941	-3.71087	1.03E-29	8.14E-29
PLOD1	22.33062	56.13844	33.80782	2.72E-31	2.65E-30
PLXDC1	0.793058	5.887244	5.094186	6.26E-38	3.43E-36
PMEPA1	8.418645	25.88378	17.46514	1.25E-24	6.15E-24
POLR1E	5.065133	8.677656	3.612522	1.46E-23	6.56E-23
POLR2K	25.27023	20.83111	-4.43912	1.89E-13	4.35E-13
PPA2	15.587	9.130783	-6.45622	3.13E-30	2.63E-29
PPIL1	16.2632	10.06174	-6.20146	1.93E-21	7.42E-21
PPIL4	12.27073	9.597034	-2.6737	6.84E-11	1.36E-10
PPM1F	3.499927	7.319641	3.819714	2.33E-27	1.43E-26
PPM1K	5.446468	1.921653	-3.52482	2.00E-36	6.04E-35
PPP1R15A	22.13926	34.79714	12.65788	2.06E-09	3.77E-09
PPP1R2	12.74418	10.02426	-2.71992	1.56E-15	4.07E-15
PPP1R3B	8.881541	22.10896	13.22742	4.54E-28	3.00E-27
PPP2R5A	24.014	16.40408	-7.60993	1.64E-23	7.33E-23
PPT1	48.14141	62.85246	14.71105	2.21E-12	4.81E-12
PREPL	17.25681	11.02517	-6.23164	6.34E-23	2.72E-22
PRF1	1.67741	10.78212	9.104709	6.58E-35	1.29E-33
PRKAR1A	64.59667	45.18233	-19.4143	2.32E-24	1.11E-23
PRKCA	8.382534	3.659702	-4.72283	9.88E-32	1.03E-30
PRPS2	27.31549	13.70809	-13.6074	5.37E-35	1.08E-33
PRR13	49.40799	35.76712	-13.6409	2.49E-20	8.83E-20
PSAT1	44.95857	7.969221	-36.9893	4.15E-24	1.94E-23
PSMA3	16.75632	18.92425	2.167929	0.000304	0.000404
PSMD8	36.71859	40.74306	4.024471	4.93E-05	6.90E-05
PTGS2	5.727144	2.505845	-3.2213	2.04E-14	4.97E-14
PTMA	165.6641	183.5953	17.93123	0.008283	0.009989
PTP4A3	5.701082	20.87827	15.17718	2.77E-34	4.68E-33
PTPRJ	11.41952	7.796345	-3.62317	1.45E-17	4.28E-17
PTPRK	9.284379	12.52408	3.239698	1.07E-07	1.76E-07
PYGL	3.956743	16.08836	12.13162	1.28E-35	3.01E-34
RAB11FIP1	6.947673	3.553344	-3.39433	8.82E-24	4.02E-23
RAB11FIP2	9.235496	5.259293	-3.9762	5.75E-27	3.43E-26
RAB20	17.10694	22.42549	5.318547	3.80E-08	6.47E-08
RAB24	2.272892	7.245195	4.972303	5.39E-33	7.12E-32

RAB5B	36.24847	27.84433	-8.40415	3.53E-29	2.63E-28
RAB5C	48.10345	33.65143	-14.452	2.46E-31	2.41E-30
RABL3	8.465092	6.084938	-2.38015	9.51E-22	3.73E-21
RALA	12.02218	15.39966	3.37748	3.66E-09	6.61E-09
RALB	32.5389	41.65778	9.118883	7.25E-12	1.53E-11
RALBP1	39.63921	14.36988	-25.2693	2.29E-38	1.49E-36
RARA	7.262339	15.19484	7.932501	8.71E-27	5.11E-26
RARS	14.34752	19.75122	5.403708	4.96E-15	1.26E-14
RASGRP3	2.125646	4.549844	2.424198	1.03E-25	5.53E-25
RCN3	4.469269	12.36702	7.89775	5.89E-19	1.90E-18
RERE	17.81663	15.20526	-2.61137	3.35E-06	5.06E-06
RGS14	4.573026	11.91387	7.340848	6.75E-26	3.69E-25
RHCG	62.55006	20.79701	-41.753	1.13E-36	3.78E-35
RHOB	189.4796	354.0616	164.582	1.51E-09	2.78E-09
RHOBTB1	10.63234	17.21264	6.580297	1.22E-08	2.13E-08
RHOG	15.55436	30.87613	15.32177	9.16E-32	9.60E-31
RMND5A	14.1932	10.34774	-3.84545	2.55E-17	7.41E-17
RNASE6	6.939145	20.28706	13.34791	2.05E-24	9.83E-24
RNF166	2.037513	5.059567	3.022054	9.34E-36	2.30E-34
RNF213	7.567684	11.51647	3.948784	1.72E-13	3.99E-13
RPL14	35.80054	43.76213	7.961583	0.000517	0.000677
RPL21	82.26761	97.51578	15.24817	0.001559	0.001981
RPL3	318.5294	459.7664	141.237	3.64E-20	1.28E-19
RPS23	93.3741	142.5272	49.1531	2.94E-21	1.11E-20
RPS25	208.1666	338.7037	130.5371	1.03E-25	5.53E-25
RPS5	75.91488	147.6936	71.77869	1.69E-28	1.17E-27
RPS6KA1	11.08876	8.493725	-2.59504	9.06E-15	2.26E-14
RTN3	42.7168	28.46734	-14.2495	2.00E-12	4.35E-12
RTN4IP1	6.136077	3.478412	-2.65766	1.06E-28	7.47E-28
RYBP	9.741331	7.724015	-2.01732	2.05E-12	4.46E-12
S100A11	287.5913	426.0247	138.4334	7.09E-07	1.12E-06
SALL2	4.833035	2.675167	-2.15787	2.30E-16	6.32E-16
SBDS	67.40811	59.62881	-7.7793	4.84E-06	7.24E-06
SBNO2	5.164889	10.42105	5.256161	3.89E-24	1.83E-23
SCRN2	18.49594	12.86885	-5.62709	0.000194	0.000261
SEC62	35.19682	27.9721	-7.22472	8.01E-17	2.26E-16
SEMA3B	11.76149	4.609254	-7.15223	1.93E-28	1.33E-27
SEPHS1	19.39759	13.20869	-6.1889	4.29E-32	4.75E-31
5-Sep	1.023569	3.399166	2.375597	2.08E-28	1.42E-27
6-Sep	9.533079	6.418354	-3.11472	1.44E-16	4.00E-16
SERPINE1	26.71965	121.9353	95.21566	1.69E-15	4.40E-15
SERPING1	114.368	176.6233	62.2553	5.60E-06	8.34E-06
SESN2	11.20349	7.653883	-3.54961	1.40E-08	2.45E-08
SETBP1	5.606583	2.984985	-2.6216	4.04E-26	2.25E-25

SFXN2	9.008744	1.860068	-7.14868	1.03E-30	9.23E-30
SGPL1	15.58231	11.60406	-3.97825	4.01E-11	8.08E-11
SH3BP2	4.034804	13.43978	9.404979	1.37E-34	2.48E-33
SH3PXD2A	3.185817	6.302985	3.117168	4.35E-20	1.52E-19
SH3YL1	9.136237	5.974206	-3.16203	4.84E-12	1.03E-11
SHISA5	26.67117	34.27096	7.599788	4.93E-13	1.11E-12
SHKBP1	10.60571	13.04182	2.436109	2.59E-10	4.98E-10
SHMT1	42.60595	21.60541	-21.0005	1.87E-15	4.87E-15
SHROOM1	1.991107	4.35139	2.360283	4.94E-24	2.30E-23
SIPA1L2	3.187093	7.282658	4.095565	4.73E-24	2.20E-23
SIRT2	7.622034	10.85283	3.230792	2.04E-29	1.56E-28
SLA	1.537339	5.43405	3.89671	3.15E-27	1.92E-26
SLC15A3	2.414404	7.561035	5.146631	2.07E-32	2.43E-31
SLC20A2	12.20491	4.655861	-7.54905	1.44E-38	9.90E-37
SLC22A4	2.625763	5.739698	3.113935	3.96E-12	8.47E-12
SLC25A37	3.379612	6.881192	3.50158	3.24E-17	9.33E-17
SLC2A3	5.57061	22.79552	17.22491	1.51E-26	8.71E-26
SLC35E1	10.22401	13.26472	3.040706	8.01E-12	1.69E-11
SLC38A1	21.18177	31.95883	10.77706	7.17E-10	1.34E-09
SLC43A2	18.68026	8.808726	-9.87154	9.68E-29	6.88E-28
SLC45A4	7.470025	4.534874	-2.93515	3.11E-16	8.47E-16
SLC5A12	37.13158	13.53986	-23.5917	0.000412	0.000543
SLC6A12	24.7042	10.77734	-13.9269	4.05E-06	6.10E-06
SLC8A1	4.233672	1.735051	-2.49862	1.47E-06	2.28E-06
SMAP2	9.756515	17.81345	8.056935	1.02E-23	4.63E-23
SMARCB1	24.05643	26.55214	2.49571	7.77E-06	1.15E-05
SNN	16.08875	10.73885	-5.3499	3.35E-21	1.26E-20
SNRPA1	3.400277	5.553816	2.153539	1.92E-25	1.01E-24
SNRPF	6.077471	8.656414	2.578944	5.30E-15	1.34E-14
SNX29	10.98676	8.788443	-2.19831	6.07E-07	9.61E-07
SOD2	39.52758	90.10934	50.58176	4.61E-15	1.17E-14
SP1	16.37865	19.51217	3.133517	6.05E-07	9.58E-07
SP2	10.56333	8.318083	-2.24524	7.46E-10	1.40E-09
SREBF1	7.018727	13.00983	5.991103	3.24E-17	9.33E-17
ST3GAL4	15.43632	6.453408	-8.98291	7.10E-25	3.55E-24
ST6GAL1	62.3045	13.36921	-48.9353	5.32E-37	2.03E-35
STAP1	9.677988	6.034139	-3.64385	1.42E-35	3.30E-34
STARD10	11.00915	8.52213	-2.48702	3.25E-12	7.00E-12
STAT3	35.12461	27.74807	-7.37654	8.41E-09	1.49E-08
STMN3	7.10587	31.17911	24.07324	1.23E-29	9.67E-29
SUSD3	10.6412	5.676373	-4.96483	4.81E-07	7.66E-07
SWAP70	11.82899	13.99422	2.165231	0.002143	0.002695
SYNJ2BP	16.61969	7.14131	-9.47838	8.85E-37	3.14E-35
SYTL3	2.178596	5.022064	2.843468	1.37E-21	5.32E-21

TAGLN2	105.3737	195.0582	89.68451	1.03E-23	4.66E-23
TCEA3	22.83494	38.91835	16.08341	3.19E-15	8.16E-15
TCEAL3	13.4679	7.836171	-5.63173	2.22E-22	9.12E-22
TCEAL4	36.84638	34.30908	-2.5373	0.003183	0.003955
TCERG1	2.762257	5.396405	2.634148	2.83E-26	1.60E-25
TCN2	47.27309	69.03048	21.75739	1.49E-05	2.16E-05
TEF	17.08668	10.90065	-6.18603	1.04E-11	2.16E-11
TFE3	14.9441	16.94884	2.004735	6.45E-06	9.57E-06
TGFB1	14.84169	43.80108	28.95939	8.28E-32	8.71E-31
THEM4	6.683804	4.162128	-2.52168	8.67E-30	6.89E-29
THRAP3	33.09527	27.97191	-5.12336	1.98E-08	3.43E-08
TLE2	7.410316	4.236705	-3.17361	2.46E-19	8.14E-19
TLR4	5.908661	8.094908	2.186246	0.00018	0.000244
TLR5	4.34232	1.913259	-2.42906	1.39E-20	5.02E-20
TMC4	26.97229	6.029732	-20.9426	1.48E-32	1.80E-31
TMCC3	6.080406	9.102006	3.021599	2.11E-05	3.04E-05
TMEM116	4.406298	1.873106	-2.53319	9.00E-33	1.14E-31
TMEM127	27.71796	23.97509	-3.74286	3.09E-06	4.69E-06
TMEM140	19.64592	52.58952	32.9436	5.76E-35	1.14E-33
TMEM164	11.35632	4.0233	-7.33302	1.44E-39	1.66E-37
TMEM177	6.2709	3.508558	-2.76234	1.40E-25	7.44E-25
TMEM204	17.61685	34.6895	17.07265	8.22E-13	1.83E-12
TMEM25	7.513849	3.245368	-4.26848	2.29E-35	5.03E-34
TMEM45B	18.58362	1.202243	-17.3814	1.09E-41	6.17E-39
TMEM8B	6.693157	3.925896	-2.76726	1.22E-25	6.53E-25
TNFAIP6	0.569658	35.32574	34.75608	5.81E-38	3.25E-36
TNFRSF1A	22.37753	43.55892	21.18138	3.59E-27	2.18E-26
TNFSF10	51.96373	83.75021	31.78648	3.53E-08	6.03E-08
TOMM20	71.08871	84.78803	13.69932	0.000543	0.000711
TOMM7	63.69615	84.25252	20.55637	6.55E-11	1.30E-10
TOP1	30.4174	25.50933	-4.90807	4.74E-11	9.51E-11
TP53I11	9.189451	19.54064	10.35119	6.23E-18	1.88E-17
TP53INP2	28.93769	26.60308	-2.33461	0.029651	0.034183
TPCN2	3.02999	5.432791	2.402801	1.17E-19	3.97E-19
TPST1	11.22283	7.297364	-3.92546	9.72E-14	2.28E-13
TRAFD1	8.540211	11.4366	2.896385	4.56E-11	9.16E-11
TRIB1	25.07911	12.34637	-12.7327	2.69E-21	1.02E-20
TRIM2	26.69296	7.629952	-19.063	2.29E-35	5.03E-34
TSC22D3	35.17361	78.12439	42.95078	2.71E-13	6.20E-13
TSPAN18	8.240334	21.2083	12.96797	4.21E-21	1.58E-20
TSPAN2	4.803767	2.782835	-2.02093	9.56E-11	1.89E-10
TSPAN3	31.09242	22.99178	-8.10064	7.97E-24	3.64E-23
TSPAN4	7.397577	17.02768	9.630099	6.56E-33	8.49E-32
TSPYL5	12.62531	4.062507	-8.5628	2.32E-31	2.28E-30

TSR1	6.515464	8.605946	2.090482	2.78E-14	6.73E-14
TTC3	13.34455	10.83521	-2.50935	6.65E-11	1.32E-10
TUBB6	7.763665	17.16057	9.396904	9.14E-24	4.16E-23
TYK2	6.574982	12.08838	5.513396	8.57E-35	1.63E-33
UBA5	7.010193	11.85592	4.845731	9.83E-16	2.60E-15
UBE2B	17.65181	23.00619	5.354383	4.93E-13	1.11E-12
UBE2C	0.82244	6.0132	5.19076	7.78E-35	1.49E-33
UBL7	21.00145	18.00526	-2.99619	3.15E-09	5.70E-09
UBTD1	15.16429	7.645875	-7.51842	9.03E-17	2.54E-16
ULK1	5.83063	8.562715	2.732086	1.17E-09	2.16E-09
UPP1	4.363011	8.78688	4.423868	1.52E-15	3.98E-15
USP4	9.706623	7.433581	-2.27304	3.64E-20	1.28E-19
VSIG4	5.905771	18.19611	12.29034	1.08E-16	3.02E-16
WAS	1.768378	8.080171	6.311793	4.20E-33	5.66E-32
WDR75	4.886879	6.903378	2.016499	3.16E-22	1.29E-21
WWP1	16.76376	12.38107	-4.38269	1.55E-19	5.22E-19
XPA	9.851556	6.495154	-3.3564	2.62E-21	9.98E-21
ZBTB7B	8.833117	12.33598	3.502865	5.05E-19	1.64E-18
ZCCHC17	16.51391	14.12851	-2.3854	3.93E-10	7.46E-10
ZDHHC18	3.164853	5.289142	2.124289	1.07E-30	9.53E-30
ZDHHC3	13.64501	6.803601	-6.84141	3.11E-33	4.27E-32
ZER1	21.46157	14.37862	-7.08296	8.53E-31	7.71E-30
ZFAND3	28.48906	22.07736	-6.4117	4.76E-21	1.77E-20
ZFYVE1	8.063127	5.994756	-2.06837	3.36E-18	1.03E-17
ZFYVE9	8.966858	5.240273	-3.72658	1.54E-30	1.35E-29
ZMAT1	1.833548	4.17636	2.342811	1.42E-11	2.94E-11
ZNF395	9.711918	61.92088	52.20896	9.34E-36	2.30E-34
ZNF787	3.904393	6.034328	2.129935	4.74E-19	1.54E-18
