# Supplementary material for: Effects and Prognostic Values of Circadian Genes CSNK1E/GNA11/KLF9/THRAP3 in Kidney Renal Clear Cell Carcinoma via a Comprehensive Analysis
Source: Bioengineering (Basel). 2022 Jul 11;9(7):306. doi: 10.3390/bioengineering9070306 (PMC9311602; doi:10.3390/bioengineering9070306)
Supplement: Supplementary file 1 [file bioengineering-09-00306-s001.zip › Supplementary Table.S2.pdf]

Supplementary Table S2.

GO analysis

ONTOLOGY	Description	P.adjust
BP	neutrophil degranulation	0.000149
BP	neutrophil activation involved in immune response	0.000149
BP	neutrophil mediated immunity	0.000149
BP	neutrophil activation	0.000149
BP	protein targeting	0.002193
BP	positive regulation of cytokine production	0.005486
BP	response to steroid hormone	0.013243
BP	negative regulation of immune system process	0.013243
BP	response to radiation	0.013243
BP	intracellular receptor signaling pathway	0.013243
BP	macrophage activation	0.013243
BP	regulation of nucleotide-binding oligomerization domain containing signaling pathway	0.013243
BP	regulation of hemopoiesis	0.013243
BP	regulation of leukocyte differentiation	0.013266
BP	alpha-beta T cell differentiation	0.014715
BP	fatty acid metabolic process	0.016456
BP	regulation of myeloid cell differentiation	0.017661
BP	mucopolysaccharide metabolic process	0.021516
BP	regulation of myeloid leukocyte differentiation	0.024857
BP	alpha-beta T cell activation	0.028965
BP	myeloid cell differentiation	0.028965
BP	circadian rhythm	0.029862
BP	positive regulation of leukocyte differentiation	0.030266
BP	positive regulation of hemopoiesis	0.030266
BP	extrinsic apoptotic signaling pathway	0.030266
BP	positive regulation of steroid biosynthetic process	0.030266
BP	negative regulation of macrophage activation	0.030266
BP	positive regulation of myeloid cell differentiation	0.032378
BP	lymphocyte mediated immunity	0.032378
BP	response to lipopolysaccharide	0.032378
BP	regulation of lipid metabolic process	0.032378
BP	regulation of pattern recognition receptor signaling pathway	0.032378
BP	negative regulation of hemopoiesis	0.032378
BP	translational initiation	0.032378
BP	rhythmic process	0.032378
BP	adaptive immune response based on somatic recombination of immune receptors built from immunoglobulin superfamily domains	0.032378
BP	serine family amino acid biosynthetic process	0.032378
BP	positive regulation of transforming growth factor beta	0.032378

	production	
BP	chondroitin sulfate metabolic process	0.032775
BP	positive regulation of interleukin-10 production	0.032775
BP	regulation of macrophage differentiation	0.036217
BP	protein transmembrane transport	0.036579
BP	cellular response to external stimulus	0.036885
BP	regulation of transforming growth factor beta production	0.037772
BP	humoral immune response	0.037772
BP	protein refolding	0.037772
BP	negative regulation of myeloid cell apoptotic process	0.037772
BP	myeloid leukocyte differentiation	0.038437
BP	response to molecule of bacterial origin	0.039642
BP	transforming growth factor beta production	0.042176
BP	chondroitin sulfate proteoglycan metabolic process	0.046184
BP	chondroitin sulfate biosynthetic process	0.046654
BP	membrane raft organization	0.046654
BP	negative regulation of response to external stimulus	0.047895
BP	negative regulation of myeloid cell differentiation	0.048573
BP	protein targeting to ER	0.048573
BP	circadian regulation of gene expression	0.048573
BP	homeostasis of number of cells	0.048573
BP	negative regulation of transport	0.048691
BP	protein targeting to peroxisome	0.048691
BP	protein localization to peroxisome	0.048691
BP	establishment of protein localization to peroxisome	0.048691
BP	protein localization to endoplasmic reticulum	0.049046
BP	L-serine metabolic process	0.049659
CC	focal adhesion	0.002905
CC	cell-substrate junction	0.002905
CC	lysosomal lumen	0.002905
CC	ficolin-1-rich granule	0.004138
CC	membrane raft	0.004207
CC	membrane microdomain	0.004207
CC	secretory granule membrane	0.006477
CC	peroxisomal matrix	0.006477
CC	microbody lumen	0.006477
CC	ficolin-1-rich granule lumen	0.006477
CC	organelle outer membrane	0.007182
CC	tertiary granule lumen	0.007182
CC	outer membrane	0.007182
CC	mitochondrial outer membrane	0.007895
CC	tertiary granule	0.007895
CC	vacuolar lumen	0.011314
CC	blood microparticle	0.01281

CC	cell-cell junction	0.044649
MF	ubiquitin-like protein ligase binding	0.044834
MF	nuclear receptor activity	0.044834
MF	ligand-activated transcription factor activity	0.044834

BP: biological process; CC: cell component; MF: molecular function
