# Supplementary material for: Effects and Prognostic Values of Circadian Genes CSNK1E/GNA11/KLF9/THRAP3 in Kidney Renal Clear Cell Carcinoma via a Comprehensive Analysis
Source: Bioengineering (Basel). 2022 Jul 11;9(7):306. doi: 10.3390/bioengineering9070306 (PMC9311602; doi:10.3390/bioengineering9070306)
Supplement: Supplementary file 1 [file bioengineering-09-00306-s001.zip › Supplementary Table.S3.pdf]

Supplementary Table S3.

The KEGG pathway enrichment analysis

ID	Description	P.adjust
hsa05417	Lipid and atherosclerosis	0.004841957
hsa05145	Toxoplasmosis	0.004841957
hsa04210	Apoptosis	0.004841957
hsa04142	Lysosome	0.009512787
hsa05171	Coronavirus disease - COVID-19	0.012288145
hsa05142	Chagas disease	0.019348948
hsa04146	Peroxisome	0.019348948
hsa04610	Complement and coagulation cascades	0.021590778
hsa05152	Tuberculosis	0.022415539
hsa00532	Glycosaminoglycan biosynthesis - chondroitin sulfate / dermatan sulfate	0.02544765
hsa05162	Measles	0.028562598
hsa05133	Pertussis	0.035832259
