# Supplementary material for: Effects and Prognostic Values of Circadian Genes CSNK1E/GNA11/KLF9/THRAP3 in Kidney Renal Clear Cell Carcinoma via a Comprehensive Analysis
Source: Bioengineering (Basel). 2022 Jul 11;9(7):306. doi: 10.3390/bioengineering9070306 (PMC9311602; doi:10.3390/bioengineering9070306)
Supplement: Supplementary file 1 [file bioengineering-09-00306-s001.zip › Supplementary Table.S4.pdf]

Supplementary Table S4.

The expression levels of DERGs in the normal and KIRC tissues.

ID	CSNK1E	GNA11	KLF9	THRAP3	Type
TCGA-B2-5641-11A-01R-1541-07	8.200851	23.28076	18.87007	40.91592	Normal
TCGA-CZ-5467-11A-01R-1503-07	7.98131	20.10002	40.11176	33.56335	Normal
TCGA-B8-5552-11A-01R-1672-07	8.276639	9.74039	35.46106	45.2991	Normal
TCGA-CZ-5455-11A-01R-1503-07	6.249228	16.52085	50.46896	36.04836	Normal
TCGA-CZ-5470-11A-01R-1503-07	7.287639	17.77508	17.56212	35.64925	Normal
TCGA-CJ-5676-11A-01R-1541-07	8.468139	14.85968	44.5875	23.23294	Normal
TCGA-CW-5580-11A-02R-1672-07	8.5812	18.9083	75.24529	36.56068	Normal
TCGA-B8-5549-11A-01R-1541-07	7.760626	20.28663	58.84236	47.64966	Normal
TCGA-CZ-4865-11A-01R-1503-07	7.869122	17.85927	25.09362	37.30003	Normal
TCGA-B0-5706-11A-01R-1541-07	10.17434	16.26611	47.74736	35.38681	Normal
TCGA-B0-5690-11A-01R-1541-07	5.973464	19.5202	18.78483	35.09357	Normal
TCGA-B8-4620-11A-01R-1758-07	9.579846	16.47719	54.80563	25.69752	Normal
TCGA-CZ-5457-11A-01R-1503-07	7.588912	20.56985	23.50573	39.20399	Normal
TCGA-CJ-5672-11A-01R-1541-07	7.74994	20.3557	31.22516	40.3813	Normal
TCGA-B0-5691-11A-01R-1541-07	7.111735	16.27085	56.90067	35.06788	Normal
TCGA-B0-5699-11A-01R-1541-07	5.779695	18.76377	43.44504	31.01661	Normal
TCGA-CZ-5468-11A-01R-1503-07	8.820951	24.90771	16.60595	34.50163	Normal
TCGA-CZ-5462-11A-01R-1503-07	5.966119	18.20965	14.74808	36.45577	Normal
TCGA-B0-5697-11A-01R-1541-07	6.630032	21.54022	31.28429	33.4257	Normal
TCGA-B0-5694-11A-01R-1541-07	9.455183	19.37598	26.48585	32.23407	Normal
TCGA-CZ-5461-11A-01R-1503-07	6.627021	14.17403	38.9154	36.39342	Normal
TCGA-B0-5696-11A-01R-1541-07	5.968307	18.23352	23.23076	33.34777	Normal
TCGA-B0-5705-11A-01R-1541-07	7.035853	16.31953	50.13819	33.06134	Normal
TCGA-CZ-5988-11A-01R-1672-07	5.951665	11.89319	51.33695	28.88932	Normal
TCGA-CZ-5984-11A-01R-1672-07	6.833934	17.62346	32.65224	28.47548	Normal
TCGA-B0-5402-11A-01R-1503-07	8.500569	16.29583	58.26614	28.23122	Normal
TCGA-CZ-5458-11A-01R-1503-07	8.564627	18.49879	65.86105	35.41113	Normal
TCGA-CZ-5469-11A-01R-1503-07	8.939133	16.847	53.27463	45.65748	Normal
TCGA-CZ-5454-11A-01R-1503-07	9.948152	16.73254	62.01754	40.55651	Normal
TCGA-CZ-5463-11A-01R-1503-07	7.51625	21.59393	43.41948	38.98843	Normal
TCGA-CJ-5681-11A-01R-1541-07	8.964407	17.96202	28.26156	31.13927	Normal
TCGA-CZ-4863-11A-01R-1503-07	6.672285	15.05836	17.14297	34.9168	Normal
TCGA-A3-3387-11A-01R-1541-07	4.401566	12.16573	47.4038	18.87129	Normal
TCGA-B2-5636-11A-01R-1541-07	6.512236	17.28266	17.78836	31.4014	Normal
TCGA-CZ-5986-11A-01R-1672-07	7.038358	13.49542	29.3083	30.8523	Normal
TCGA-CJ-5689-11A-01R-1541-07	10.94515	19.81258	32.02372	38.69643	Normal
TCGA-B0-5711-11A-01R-1672-07	7.529855	15.997	31.94432	40.35073	Normal
TCGA-CW-5585-11A-01R-1541-07	6.517995	19.38217	41.31248	29.64985	Normal
TCGA-CZ-5451-11A-01R-1503-07	5.759695	17.29177	45.22948	28.97114	Normal
TCGA-CZ-5982-11A-01R-1672-07	4.235611	14.5637	54.37833	25.21369	Normal
TCGA-CZ-5465-11A-01R-1503-07	8.542989	21.43844	22.30703	40.08595	Normal

TCGA-CW-5584-11A-01R-1541-07	9.911833	19.03534	19.6871	40.3589	Normal
TCGA-CJ-6033-11A-01R-1858-07	8.63797	17.0641	17.43467	30.23335	Normal
TCGA-CJ-5680-11A-01R-1541-07	16.10212	18.56622	26.81498	39.51652	Normal
TCGA-B0-4712-11A-02R-1503-07	8.444621	19.67959	18.64651	45.30611	Normal
TCGA-B0-5703-11A-01R-1541-07	9.040364	13.90006	32.31121	28.65326	Normal
TCGA-B0-5712-11A-01R-1672-07	11.0956	16.47923	45.98981	35.04885	Normal
TCGA-CZ-5989-11A-01R-1672-07	10.96492	18.15407	20.67531	43.51735	Normal
TCGA-A3-3358-11A-01R-1541-07	4.789163	11.58383	31.66857	20.80957	Normal
TCGA-CZ-5987-11A-01R-1672-07	9.194493	12.99497	38.31609	35.89592	Normal
TCGA-CJ-6030-11A-01R-1672-07	6.1125	14.59991	30.91511	25.4117	Normal
TCGA-CW-5581-11A-01R-1541-07	6.091654	18.97004	32.6594	31.68061	Normal
TCGA-CZ-5456-11A-02R-1503-07	9.33913	20.77696	48.25356	33.53102	Normal
TCGA-CJ-5677-11A-01R-1541-07	7.130073	18.2533	21.58389	30.28712	Normal
TCGA-B0-5709-11A-01R-1541-07	7.26128	14.73291	28.70949	31.5878	Normal
TCGA-B0-4700-11A-01R-1541-07	7.686941	15.80978	24.91732	23.52292	Normal
TCGA-CJ-5679-11A-01R-1541-07	9.539412	18.28782	25.57521	32.44012	Normal
TCGA-CW-5591-11A-01R-1541-07	11.93275	14.25436	14.62104	26.4869	Normal
TCGA-CJ-5678-11A-01R-1541-07	5.209366	15.70912	20.08998	24.80466	Normal
TCGA-CZ-5985-11A-01R-1672-07	4.764532	7.953561	55.00626	27.85052	Normal
TCGA-CW-6090-11A-01R-1672-07	4.900191	12.04767	22.56633	29.89747	Normal
TCGA-CW-5589-11A-01R-1541-07	6.162663	16.88759	36.36864	35.18222	Normal
TCGA-CW-5587-11A-01R-1541-07	4.645625	15.77826	20.38657	36.16406	Normal
TCGA-CZ-5452-11A-01R-1503-07	8.650181	22.16214	24.43285	33.21855	Normal
TCGA-CW-6088-11A-01R-1672-07	7.101418	19.30451	27.86631	42.62425	Normal
TCGA-CZ-5466-11A-01R-1503-07	5.640437	13.61267	26.87546	28.29014	Normal
TCGA-B0-5701-11A-01R-1541-07	8.984716	19.53727	58.12851	34.54678	Normal
TCGA-B8-4619-11A-01R-1758-07	4.378968	11.00701	46.75758	17.0739	Normal
TCGA-CZ-5453-11A-01R-1503-07	8.218431	19.76054	33.11137	37.84386	Normal
TCGA-CZ-4864-11A-01R-1503-07	4.904772	13.70675	40.76839	22.622	Normal
TCGA-B8-4622-11A-01R-1758-07	4.647518	12.28946	33.08386	16.43047	Normal
TCGA-CW-6087-11A-01R-1672-07	5.139926	12.48018	21.11464	28.1775	Normal
TCGA-CJ-6033-01A-11R-1672-07	17.44806	12.5952	15.11387	25.0074	Tumor
TCGA-B0-5706-01A-11R-1541-07	22.24121	9.868628	23.92232	30.42275	Tumor
TCGA-CW-5587-01A-01R-1541-07	7.78657	14.31081	22.34751	37.20058	Tumor
TCGA-CZ-4864-01A-01R-1503-07	12.25581	11.07817	44.14703	25.74025	Tumor
TCGA-B2-5636-01A-02R-1541-07	15.14181	21.16055	21.9347	34.69372	Tumor
TCGA-CZ-5987-01A-11R-1672-07	14.17599	8.573845	15.80577	24.74942	Tumor
TCGA-CZ-5468-01A-01R-1503-07	11.40868	11.07766	15.34892	23.15447	Tumor
TCGA-CW-6090-01A-11R-1672-07	14.76876	6.906149	15.25174	30.42856	Tumor
TCGA-B0-4700-01A-02R-1541-07	18.6853	8.937563	19.10088	23.95656	Tumor
TCGA-B0-5705-01A-11R-1541-07	14.38635	13.42315	37.31432	32.79666	Tumor
TCGA-CZ-5984-01A-11R-1672-07	9.582389	9.087706	14.68549	24.25699	Tumor
TCGA-CJ-5678-01A-11R-1541-07	7.487363	7.549766	15.37718	17.7321	Tumor
TCGA-CZ-5467-01A-01R-1503-07	15.63999	15.71857	68.28659	38.84928	Tumor

TCGA-B0-5690-01A-11R-1541-07	22.79403	19.54458	27.53565	36.35623	Tumor
TCGA-CZ-5452-01A-01R-1503-07	12.98166	10.12779	11.80459	28.22022	Tumor
TCGA-CZ-4865-01A-02R-1503-07	17.37491	14.68549	19.77904	36.29934	Tumor
TCGA-CZ-5453-01A-01R-1503-07	18.00342	17.31306	29.142	26.2954	Tumor
TCGA-B8-4619-01A-02R-1325-07	11.03643	39.07485	45.79338	19.16131	Tumor
TCGA-CJ-5676-01A-11R-1541-07	16.92515	10.18955	21.87654	20.59751	Tumor
TCGA-B0-5703-01A-11R-1541-07	13.07516	14.23707	42.17841	17.68782	Tumor
TCGA-B0-5694-01A-11R-1541-07	15.47297	9.817271	14.19227	25.22591	Tumor
TCGA-B0-5711-01A-11R-1672-07	12.80824	11.68401	33.0402	37.79452	Tumor
TCGA-CJ-5689-01A-11R-1541-07	13.71226	12.97709	24.6975	25.71975	Tumor
TCGA-CZ-5986-01A-11R-1672-07	12.93177	6.347526	34.43361	38.75841	Tumor
TCGA-CZ-5458-01A-01R-1503-07	14.36833	13.82999	52.02622	31.48245	Tumor
TCGA-A3-3387-01A-01R-1541-07	13.51763	10.31785	33.57329	30.2196	Tumor
TCGA-CZ-5465-01A-01R-1503-07	29.49574	9.386246	24.28698	42.95032	Tumor
TCGA-B8-5549-01A-01R-1541-07	8.787499	16.4312	42.2397	26.37845	Tumor
TCGA-CW-5585-01A-01R-1541-07	12.76567	19.79447	56.50501	36.49423	Tumor
TCGA-CZ-5469-01A-01R-1503-07	13.78127	9.662187	16.18244	11.97004	Tumor
TCGA-B0-5699-01A-11R-1541-07	14.32529	13.20961	31.11971	41.52119	Tumor
TCGA-B8-4622-01A-02R-1277-07	15.87268	10.35121	24.90062	28.7691	Tumor
TCGA-B8-5552-01B-11R-1672-07	14.73515	17.10508	35.81877	36.08905	Tumor
TCGA-CJ-5677-01A-11R-1541-07	12.55859	10.00209	7.496649	17.95047	Tumor
TCGA-CZ-4863-01A-01R-1503-07	12.06066	13.14788	30.94017	30.72679	Tumor
TCGA-B0-5691-01A-11R-1541-07	12.4203	19.58848	85.44491	37.83965	Tumor
TCGA-B2-5641-01A-01R-1541-07	10.43229	12.13997	9.40661	30.62533	Tumor
TCGA-B0-5709-01A-11R-1541-07	13.86616	16.79401	35.64279	30.82362	Tumor
TCGA-CW-6087-01A-11R-1672-07	8.734051	7.428645	6.593661	36.85149	Tumor
TCGA-CZ-5988-01A-11R-1672-07	16.74078	6.344174	45.74824	35.73969	Tumor
TCGA-CW-5591-01A-01R-1541-07	9.847636	20.96963	14.55527	40.0725	Tumor
TCGA-CZ-5470-01A-01R-1503-07	8.877605	7.95764	15.4009	26.85256	Tumor
TCGA-B0-5402-01A-01R-1503-07	14.2158	9.239281	51.43322	41.70718	Tumor
TCGA-CW-5584-01A-01R-1541-07	13.33498	19.28978	23.29167	33.61886	Tumor
TCGA-CW-5589-01A-01R-1541-07	11.77195	15.72443	50.28699	34.93869	Tumor
TCGA-B0-5701-01A-11R-1541-07	5.742902	12.33671	17.84854	25.52251	Tumor
TCGA-CZ-5989-01A-11R-1672-07	4.21325	1.056843	61.8545	30.85071	Tumor
TCGA-CZ-5454-01A-01R-1503-07	9.191559	6.454781	27.9149	29.44733	Tumor
TCGA-CJ-5679-01A-11R-1541-07	12.53679	8.570187	13.95426	14.62141	Tumor
TCGA-CZ-5461-01A-01R-1503-07	11.29654	14.17786	19.6552	31.6479	Tumor
TCGA-B0-4712-01A-01R-1503-07	8.690223	6.530278	11.93224	16.81082	Tumor
TCGA-CW-6088-01A-11R-1672-07	21.72833	14.27134	40.98239	42.33203	Tumor
TCGA-CZ-5982-01A-11R-1672-07	17.41574	14.28458	39.89197	34.06878	Tumor
TCGA-CW-5580-01A-01R-1672-07	11.64233	9.205931	45.49045	36.86224	Tumor
TCGA-B0-5696-01A-11R-1541-07	15.75471	14.05231	22.6307	25.16227	Tumor
TCGA-CZ-5455-01A-01R-1503-07	9.726066	10.19172	48.96944	40.24746	Tumor
TCGA-CZ-5451-01A-01R-1503-07	12.94746	15.16322	60.65176	24.97695	Tumor

TCGA-CJ-5681-01A-11R-1541-07	12.56301	9.848428	10.61927	35.62097	Tumor
TCGA-B8-4620-01A-02R-1325-07	23.92161	9.272711	28.55303	27.10594	Tumor
TCGA-CZ-5985-01A-11R-1672-07	8.906691	7.442531	18.13616	33.73848	Tumor
TCGA-CW-5581-01A-02R-1541-07	21.57706	14.86998	24.19548	30.41702	Tumor
TCGA-A3-3358-01A-01R-1541-07	10.25725	7.623357	28.83625	23.12184	Tumor
TCGA-CJ-5680-01A-11R-1541-07	13.59305	15.04181	16.33047	35.64642	Tumor
TCGA-CZ-5463-01A-01R-1503-07	10.11862	13.00901	33.18711	33.86939	Tumor
TCGA-CZ-5466-01A-01R-1503-07	11.19341	11.57632	12.51595	22.43438	Tumor
TCGA-CJ-6030-01A-11R-1672-07	15.8015	8.202332	18.06704	33.66194	Tumor
TCGA-B0-5697-01A-11R-1541-07	11.23888	12.13463	16.91208	28.2204	Tumor
TCGA-CZ-5462-01A-01R-1503-07	8.515887	10.07762	9.366032	24.41867	Tumor
TCGA-CZ-5456-01A-01R-1503-07	11.29513	9.045019	8.527638	25.96406	Tumor
TCGA-CJ-5672-01A-11R-1541-07	10.3444	12.42149	17.39823	24.65822	Tumor
TCGA-B0-5712-01A-11R-1672-07	12.8914	10.48443	22.47344	24.60394	Tumor
TCGA-CZ-5457-01A-01R-1503-07	8.087824	13.74325	42.24481	25.39984	Tumor
