# Supplementary material for: Effects and Prognostic Values of Circadian Genes CSNK1E/GNA11/KLF9/THRAP3 in Kidney Renal Clear Cell Carcinoma via a Comprehensive Analysis
Source: Bioengineering (Basel). 2022 Jul 11;9(7):306. doi: 10.3390/bioengineering9070306 (PMC9311602; doi:10.3390/bioengineering9070306)
Supplement: Supplementary file 1 [file bioengineering-09-00306-s001.zip › Supplementary Table.S5.pdf]

Supplementary Table.S5

The clinical data of KIRC patients

Id	futime	fustat	age	gender	grade	stage	T	M
TCGA-EU-5904	551	0	47	FEMALE	G1	Stage I	T1	M0
TCGA-B4-5834	38	0	59	MALE	G1	Stage I	T1	M0
TCGA-CZ-4859	1787	0	59	FEMALE	G2	Stage I	T1	M0
TCGA-B4-5835	16	0	64	FEMALE	G2	Stage I	T1	M0
TCGA-B0-5705	3668	0	65	FEMALE	G2	Stage I	T1	M0
TCGA-A3-3331	1257	0	86	FEMALE	G2	Stage I	T1	M0
TCGA-B0-5104	0	1	90	FEMALE	G2	Stage I	T1	M0
TCGA-B4-5843	11	0	45	MALE	G2	Stage I	T1	M0
TCGA-A3-3383	861	0	52	MALE	G2	Stage I	T1	M0
TCGA-B0-5699	2741	0	53	MALE	G2	Stage I	T1	M0
TCGA-A3-3380	567	0	54	MALE	G2	Stage I	T1	M0
TCGA-A3-3311	1191	1	57	MALE	G2	Stage I	T1	M0
TCGA-BP-4353	375	1	61	MALE	G2	Stage I	T1	M0
TCGA-B4-5378	175	0	62	MALE	G2	Stage I	T1	M0
TCGA-AK-3425	3343	0	68	MALE	G2	Stage I	T1	M0
TCGA-B0-5098	1584	1	53	FEMALE	G3	Stage I	T1	M0
TCGA-EU-5905	119	0	67	FEMALE	G3	Stage I	T1	M0
TCGA-B0-5102	2764	1	74	FEMALE	G3	Stage I	T1	M0
TCGA-CZ-4866	1768	0	79	FEMALE	G3	Stage I	T1	M0
TCGA-A3-3378	630	0	60	MALE	G3	Stage I	T1	M0
TCGA-CZ-5986	373	0	61	MALE	G3	Stage I	T1	M0
TCGA-A3-3336	1043	0	75	FEMALE	G4	Stage I	T1	M0
TCGA-AS-3778	43	0	35	MALE	G1	Stage I	T1a	M0
TCGA-A3-3326	1137	0	47	MALE	G1	Stage I	T1a	M0
TCGA-CW-6093	3146	0	73	MALE	G1	Stage I	T1a	M0
TCGA-DV-5569	355	0	29	FEMALE	G2	Stage I	T1a	M0
TCGA-DV-5567	910	0	40	FEMALE	G2	Stage I	T1a	M0
TCGA-CW-6096	2701	0	44	FEMALE	G2	Stage I	T1a	M0
TCGA-BP-5174	2257	0	45	FEMALE	G2	Stage I	T1a	M0
TCGA-A3-3385	1993	0	46	FEMALE	G2	Stage I	T1a	M0
TCGA-BP-5186	693	0	50	FEMALE	G2	Stage I	T1a	M0
TCGA-CW-5583	2489	0	51	FEMALE	G2	Stage I	T1a	M0
TCGA-DV-5575	1006	0	52	FEMALE	G2	Stage I	T1a	M0
TCGA-BP-4964	1862	0	54	FEMALE	G2	Stage I	T1a	M0
TCGA-BP-4782	354	0	55	FEMALE	G2	Stage I	T1a	M0
TCGA-BP-4775	1843	0	55	FEMALE	G2	Stage I	T1a	M0
TCGA-DV-5576	727	1	55	FEMALE	G2	Stage I	T1a	M0
TCGA-A3-3358	1307	0	57	FEMALE	G2	Stage I	T1a	M0
TCGA-BP-4774	1885	0	57	FEMALE	G2	Stage I	T1a	M0
TCGA-CZ-5982	2042	0	59	FEMALE	G2	Stage I	T1a	M0
TCGA-A3-3362	1559	0	60	FEMALE	G2	Stage I	T1a	M0

TCGA-CJ-4905	1496	0	62	FEMALE	G2	Stage I	T1a	M0
TCGA-BP-4969	1794	0	63	FEMALE	G2	Stage I	T1a	M0
TCGA-DV-5566	1398	0	67	FEMALE	G2	Stage I	T1a	M0
TCGA-BP-4784	1854	0	67	FEMALE	G2	Stage I	T1a	M0
TCGA-BP-4349	372	0	68	FEMALE	G2	Stage I	T1a	M0
TCGA-CZ-4865	166	1	70	FEMALE	G2	Stage I	T1a	M0
TCGA-B0-5110	1092	0	71	FEMALE	G2	Stage I	T1a	M0
TCGA-BP-4768	400	0	72	FEMALE	G2	Stage I	T1a	M0
TCGA-B0-5120	493	0	72	FEMALE	G2	Stage I	T1a	M0
TCGA-B8-4154	255	0	73	FEMALE	G2	Stage I	T1a	M0
TCGA-BP-4795	620	0	74	FEMALE	G2	Stage I	T1a	M0
TCGA-BP-4344	1666	0	75	FEMALE	G2	Stage I	T1a	M0
TCGA-B0-4945	2145	1	75	FEMALE	G2	Stage I	T1a	M0
TCGA-CW-5588	2017	0	78	FEMALE	G2	Stage I	T1a	M0
TCGA-BP-5176	1590	1	78	FEMALE	G2	Stage I	T1a	M0
TCGA-BP-4763	1270	1	79	FEMALE	G2	Stage I	T1a	M0
TCGA-A3-3359	2504	0	82	FEMALE	G2	Stage I	T1a	M0
TCGA-AK-3450	1508	0	85	FEMALE	G2	Stage I	T1a	M0
TCGA-DV-5568	370	0	26	MALE	G2	Stage I	T1a	M0
TCGA-DV-5574	723	0	37	MALE	G2	Stage I	T1a	M0
TCGA-CJ-4908	1531	0	38	MALE	G2	Stage I	T1a	M0
TCGA-BP-5194	408	0	39	MALE	G2	Stage I	T1a	M0
TCGA-BP-4758	2208	0	40	MALE	G2	Stage I	T1a	M0
TCGA-DV-5573	1130	0	41	MALE	G2	Stage I	T1a	M0
TCGA-B8-5545	522	0	42	MALE	G2	Stage I	T1a	M0
TCGA-B8-5165	8	0	43	MALE	G2	Stage I	T1a	M0
TCGA-BP-4765	2184	0	43	MALE	G2	Stage I	T1a	M0
TCGA-A3-3365	873	0	46	MALE	G2	Stage I	T1a	M0
TCGA-BP-5008	1071	0	46	MALE	G2	Stage I	T1a	M0
TCGA-BP-4965	1871	0	46	MALE	G2	Stage I	T1a	M0
TCGA-BP-4961	1935	0	47	MALE	G2	Stage I	T1a	M0
TCGA-BP-4789	1489	0	48	MALE	G2	Stage I	T1a	M0
TCGA-A3-3387	617	0	49	MALE	G2	Stage I	T1a	M0
TCGA-B0-5697	1835	0	50	MALE	G2	Stage I	T1a	M0
TCGA-BP-4759	2372	0	50	MALE	G2	Stage I	T1a	M0
TCGA-A3-3322	1478	0	51	MALE	G2	Stage I	T1a	M0
TCGA-A3-3376	1696	1	51	MALE	G2	Stage I	T1a	M0
TCGA-BP-4776	411	0	52	MALE	G2	Stage I	T1a	M0
TCGA-CW-5589	2378	0	52	MALE	G2	Stage I	T1a	M0
TCGA-A3-3325	1170	1	52	MALE	G2	Stage I	T1a	M0
TCGA-BP-4331	2454	1	52	MALE	G2	Stage I	T1a	M0
TCGA-BP-5196	1018	0	53	MALE	G2	Stage I	T1a	M0
TCGA-BP-5180	2263	0	53	MALE	G2	Stage I	T1a	M0
TCGA-BP-5187	406	0	54	MALE	G2	Stage I	T1a	M0

TCGA-BP-4991	1413	0	54	MALE	G2	Stage I	T1a	M0
TCGA-BP-5170	2412	0	55	MALE	G2	Stage I	T1a	M0
TCGA-BP-4999	1266	0	56	MALE	G2	Stage I	T1a	M0
TCGA-BP-4801	1124	0	57	MALE	G2	Stage I	T1a	M0
TCGA-B8-4619	19	0	58	MALE	G2	Stage I	T1a	M0
TCGA-AK-3460	951	0	58	MALE	G2	Stage I	T1a	M0
TCGA-BP-5192	714	0	59	MALE	G2	Stage I	T1a	M0
TCGA-DV-5565	1329	0	59	MALE	G2	Stage I	T1a	M0
TCGA-BP-5006	840	0	61	MALE	G2	Stage I	T1a	M0
TCGA-BP-4769	1876	0	63	MALE	G2	Stage I	T1a	M0
TCGA-B0-5106	1598	1	64	MALE	G2	Stage I	T1a	M0
TCGA-BP-4177	1670	0	65	MALE	G2	Stage I	T1a	M0
TCGA-3Z-A93Z	385	0	69	MALE	G2	Stage I	T1a	M0
TCGA-BP-4760	2361	0	69	MALE	G2	Stage I	T1a	M0
TCGA-AK-3461	853	0	72	MALE	G2	Stage I	T1a	M0
TCGA-BP-4988	828	1	72	MALE	G2	Stage I	T1a	M0
TCGA-B2-5635	315	0	74	MALE	G2	Stage I	T1a	M0
TCGA-BP-5195	749	0	75	MALE	G2	Stage I	T1a	M0
TCGA-BP-5173	62	1	75	MALE	G2	Stage I	T1a	M0
TCGA-BP-5168	1463	1	75	MALE	G2	Stage I	T1a	M0
TCGA-BP-4790	1111	1	76	MALE	G2	Stage I	T1a	M0
TCGA-B0-5700	1082	0	77	MALE	G2	Stage I	T1a	M0
TCGA-B2-5636	265	0	79	MALE	G2	Stage I	T1a	M0
TCGA-CZ-4853	774	0	82	MALE	G2	Stage I	T1a	M0
TCGA-B0-4823	454	1	88	MALE	G2	Stage I	T1a	M0
TCGA-B0-5707	2828	0	39	FEMALE	G3	Stage I	T1a	M0
TCGA-CJ-4886	1952	0	42	FEMALE	G3	Stage I	T1a	M0
TCGA-BP-4766	1462	0	43	FEMALE	G3	Stage I	T1a	M0
TCGA-BP-5177	293	0	46	FEMALE	G3	Stage I	T1a	M0
TCGA-B0-4824	1657	1	49	FEMALE	G3	Stage I	T1a	M0
TCGA-B8-5159	240	0	61	FEMALE	G3	Stage I	T1a	M0
TCGA-B8-4148	379	0	63	FEMALE	G3	Stage I	T1a	M0
TCGA-B0-5691	3431	0	66	FEMALE	G3	Stage I	T1a	M0
TCGA-BP-4807	211	0	42	MALE	G3	Stage I	T1a	M0
TCGA-BP-4762	1343	1	42	MALE	G3	Stage I	T1a	M0
TCGA-BP-4777	1731	0	46	MALE	G3	Stage I	T1a	M0
TCGA-BP-4998	932	0	49	MALE	G3	Stage I	T1a	M0
TCGA-B0-4834	2090	1	49	MALE	G3	Stage I	T1a	M0
TCGA-CJ-5671	1943	0	51	MALE	G3	Stage I	T1a	M0
TCGA-BP-5004	1126	0	53	MALE	G3	Stage I	T1a	M0
TCGA-BP-5184	1133	0	54	MALE	G3	Stage I	T1a	M0
TCGA-BP-4994	1308	0	54	MALE	G3	Stage I	T1a	M0
TCGA-BP-5185	1132	0	56	MALE	G3	Stage I	T1a	M0
TCGA-BP-5182	1165	0	56	MALE	G3	Stage I	T1a	M0

TCGA-BP-4993	177	0	58	MALE	G3	Stage I	T1a	M0
TCGA-AK-3440	1745	0	58	MALE	G3	Stage I	T1a	M0
TCGA-BP-5175	932	0	60	MALE	G3	Stage I	T1a	M0
TCGA-BP-5190	1011	0	61	MALE	G3	Stage I	T1a	M0
TCGA-B0-5083	1045	1	63	MALE	G3	Stage I	T1a	M0
TCGA-CJ-6030	2299	1	65	MALE	G3	Stage I	T1a	M0
TCGA-BP-4986	785	0	75	MALE	G3	Stage I	T1a	M0
TCGA-BP-4976	1632	0	77	MALE	G3	Stage I	T1a	M0
TCGA-B0-5077	1317	1	77	MALE	G3	Stage I	T1a	M0
TCGA-BP-4781	2080	0	78	MALE	G3	Stage I	T1a	M0
TCGA-B2-5641	324	0	79	MALE	G3	Stage I	T1a	M0
TCGA-B2-4099	374	0	83	MALE	G3	Stage I	T1a	M0
TCGA-CJ-5672	1972	1	84	MALE	G3	Stage I	T1a	M0
TCGA-CJ-4889	1946	0	63	FEMALE	G4	Stage I	T1a	M0
TCGA-CJ-6027	1855	0	77	MALE	G4	Stage I	T1a	M0
TCGA-BP-4970	433	0	44	MALE	G3	Stage III	T1a	M0
TCGA-A3-3320	1508	0	52	FEMALE	G1	Stage I	T1b	M0
TCGA-B0-5690	2408	0	53	FEMALE	G1	Stage I	T1b	M0
TCGA-BP-4165	3037	0	64	FEMALE	G1	Stage I	T1b	M0
TCGA-A3-3323	1106	0	53	MALE	G1	Stage I	T1b	M0
TCGA-A3-3349	1385	0	34	FEMALE	G2	Stage I	T1b	M0
TCGA-B8-5546	505	0	38	FEMALE	G2	Stage I	T1b	M0
TCGA-B8-5552	392	0	41	FEMALE	G2	Stage I	T1b	M0
TCGA-B8-4146	511	0	41	FEMALE	G2	Stage I	T1b	M0
TCGA-BP-4987	1124	0	41	FEMALE	G2	Stage I	T1b	M0
TCGA-BP-5001	1177	0	43	FEMALE	G2	Stage I	T1b	M0
TCGA-B0-5693	3076	0	47	FEMALE	G2	Stage I	T1b	M0
TCGA-A3-3370	2274	0	48	FEMALE	G2	Stage I	T1b	M0
TCGA-A3-3374	1314	0	51	FEMALE	G2	Stage I	T1b	M0
TCGA-BP-4326	1625	1	53	FEMALE	G2	Stage I	T1b	M0
TCGA-BP-5181	1495	0	58	FEMALE	G2	Stage I	T1b	M0
TCGA-CJ-4634	1820	0	60	FEMALE	G2	Stage I	T1b	M0
TCGA-B0-5119	59	0	61	FEMALE	G2	Stage I	T1b	M0
TCGA-B4-5836	141	0	61	FEMALE	G2	Stage I	T1b	M0
TCGA-B0-5695	1420	0	61	FEMALE	G2	Stage I	T1b	M0
TCGA-CZ-4856	18	0	62	FEMALE	G2	Stage I	T1b	M0
TCGA-BP-4756	374	0	62	FEMALE	G2	Stage I	T1b	M0
TCGA-BP-4325	2964	0	64	FEMALE	G2	Stage I	T1b	M0
TCGA-CJ-4920	139	1	64	FEMALE	G2	Stage I	T1b	M0
TCGA-CJ-4892	1521	0	65	FEMALE	G2	Stage I	T1b	M0
TCGA-BP-4162	3074	0	65	FEMALE	G2	Stage I	T1b	M0
TCGA-B8-5553	435	0	67	FEMALE	G2	Stage I	T1b	M0
TCGA-BP-4340	562	1	70	FEMALE	G2	Stage I	T1b	M0
TCGA-B2-4098	51	1	72	FEMALE	G2	Stage I	T1b	M0

TCGA-BP-4170	2343	1	72	FEMALE	G2	Stage I	T1b	M0
TCGA-AK-3444	1471	0	80	FEMALE	G2	Stage I	T1b	M0
TCGA-B0-4839	1639	1	80	FEMALE	G2	Stage I	T1b	M0
TCGA-B0-4833	2386	1	82	FEMALE	G2	Stage I	T1b	M0
TCGA-CZ-5988	693	0	38	MALE	G2	Stage I	T1b	M0
TCGA-B0-5117	535	0	40	MALE	G2	Stage I	T1b	M0
TCGA-CJ-4899	1528	0	42	MALE	G2	Stage I	T1b	M0
TCGA-B0-5399	652	0	46	MALE	G2	Stage I	T1b	M0
TCGA-CZ-4862	1843	0	46	MALE	G2	Stage I	T1b	M0
TCGA-EU-5906	206	0	55	MALE	G2	Stage I	T1b	M0
TCGA-B2-5633	358	0	56	MALE	G2	Stage I	T1b	M0
TCGA-B0-5121	554	0	56	MALE	G2	Stage I	T1b	M0
TCGA-B0-5710	1459	0	57	MALE	G2	Stage I	T1b	M0
TCGA-BP-4804	1459	0	59	MALE	G2	Stage I	T1b	M0
TCGA-CW-6088	3222	0	60	MALE	G2	Stage I	T1b	M0
TCGA-B2-4102	202	0	61	MALE	G2	Stage I	T1b	M0
TCGA-BP-4176	1955	0	64	MALE	G2	Stage I	T1b	M0
TCGA-CZ-4854	1404	1	68	MALE	G2	Stage I	T1b	M0
TCGA-BP-4158	3377	0	69	MALE	G2	Stage I	T1b	M0
TCGA-A3-3319	1130	0	70	MALE	G2	Stage I	T1b	M0
TCGA-BP-4159	2601	1	70	MALE	G2	Stage I	T1b	M0
TCGA-B0-5702	1605	0	71	MALE	G2	Stage I	T1b	M0
TCGA-AK-3434	2087	0	72	MALE	G2	Stage I	T1b	M0
TCGA-B2-3924	371	0	73	MALE	G2	Stage I	T1b	M0
TCGA-A3-3329	1624	0	75	MALE	G2	Stage I	T1b	M0
TCGA-A3-3328	1385	0	79	MALE	G2	Stage I	T1b	M0
TCGA-A3-3373	1621	0	54	FEMALE	G3	Stage I	T1b	M0
TCGA-CJ-5686	2038	0	59	FEMALE	G3	Stage I	T1b	M0
TCGA-B8-5551	15	0	65	FEMALE	G3	Stage I	T1b	M0
TCGA-B0-4838	834	1	69	FEMALE	G3	Stage I	T1b	M0
TCGA-CJ-4874	2283	0	73	FEMALE	G3	Stage I	T1b	M0
TCGA-CJ-4893	750	0	76	FEMALE	G3	Stage I	T1b	M0
TCGA-BP-5000	563	0	40	MALE	G3	Stage I	T1b	M0
TCGA-BP-4975	1433	0	40	MALE	G3	Stage I	T1b	M0
TCGA-BP-4968	1746	0	40	MALE	G3	Stage I	T1b	M0
TCGA-BP-4982	1014	0	42	MALE	G3	Stage I	T1b	M0
TCGA-BP-4338	2859	0	43	MALE	G3	Stage I	T1b	M0
TCGA-CW-5581	2799	0	44	MALE	G3	Stage I	T1b	M0
TCGA-AK-3458	1168	0	48	MALE	G3	Stage I	T1b	M0
TCGA-CJ-4635	1416	0	48	MALE	G3	Stage I	T1b	M0
TCGA-BP-4959	2660	0	49	MALE	G3	Stage I	T1b	M0
TCGA-CJ-4903	1560	0	50	MALE	G3	Stage I	T1b	M0
TCGA-A3-3324	1186	0	51	MALE	G3	Stage I	T1b	M0
TCGA-CZ-5984	1491	0	51	MALE	G3	Stage I	T1b	M0

TCGA-BP-5009	1092	1	52	MALE	G3	Stage I	T1b	M0
TCGA-B8-5549	194	0	53	MALE	G3	Stage I	T1b	M0
TCGA-B0-5812	2963	0	53	MALE	G3	Stage I	T1b	M0
TCGA-B0-5088	563	1	53	MALE	G3	Stage I	T1b	M0
TCGA-CJ-6031	1906	0	54	MALE	G3	Stage I	T1b	M0
TCGA-BP-4977	454	0	57	MALE	G3	Stage I	T1b	M0
TCGA-A3-3313	735	1	59	MALE	G3	Stage I	T1b	M0
TCGA-B8-4621	431	0	63	MALE	G3	Stage I	T1b	M0
TCGA-BP-4963	1834	0	63	MALE	G3	Stage I	T1b	M0
TCGA-B0-4837	1378	1	63	MALE	G3	Stage I	T1b	M0
TCGA-A3-3306	1120	0	67	MALE	G3	Stage I	T1b	M0
TCGA-BP-4995	1371	0	68	MALE	G3	Stage I	T1b	M0
TCGA-CW-6090	2552	0	68	MALE	G3	Stage I	T1b	M0
TCGA-A3-3346	137	1	68	MALE	G3	Stage I	T1b	M0
TCGA-A3-3382	574	0	69	MALE	G3	Stage I	T1b	M0
TCGA-A3-3367	2270	0	72	MALE	G3	Stage I	T1b	M0
TCGA-B0-5703	1203	0	73	MALE	G3	Stage I	T1b	M0
TCGA-BP-4161	2746	0	74	MALE	G3	Stage I	T1b	M0
TCGA-B0-5698	2583	0	77	MALE	G3	Stage I	T1b	M0
TCGA-CJ-5683	1889	0	78	MALE	G3	Stage I	T1b	M0
TCGA-AK-3454	874	0	84	MALE	G3	Stage I	T1b	M0
TCGA-CJ-4872	1435	0	51	MALE	G4	Stage I	T1b	M0
TCGA-BP-5189	822	1	60	MALE	G4	Stage I	T1b	M0
TCGA-BP-4992	501	0	66	MALE	G4	Stage I	T1b	M0
TCGA-BP-5169	193	0	70	MALE	G4	Stage I	T1b	M0
TCGA-CJ-5689	0	1	90	MALE	G4	Stage I	T1b	M0
TCGA-A3-3347	1610	1	76	FEMALE	G2	Stage III	T1b	M0
TCGA-B4-5844	7	0	61	FEMALE	G1	Stage II	T2	M0
TCGA-CJ-4639	2308	0	49	FEMALE	G2	Stage II	T2	M0
TCGA-AK-3429	2017	0	54	FEMALE	G2	Stage II	T2	M0
TCGA-AK-3453	1397	0	58	FEMALE	G2	Stage II	T2	M0
TCGA-BP-4327	109	1	75	FEMALE	G2	Stage II	T2	M0
TCGA-BP-4169	701	1	76	FEMALE	G2	Stage II	T2	M0
TCGA-B0-4852	1121	1	78	FEMALE	G2	Stage II	T2	M0
TCGA-CZ-5469	946	1	41	MALE	G2	Stage II	T2	M0
TCGA-BP-5007	1140	0	45	MALE	G2	Stage II	T2	M0
TCGA-B0-5706	2414	0	45	MALE	G2	Stage II	T2	M0
TCGA-CJ-4642	1628	0	47	MALE	G2	Stage II	T2	M0
TCGA-A3-3363	319	0	50	MALE	G2	Stage II	T2	M0
TCGA-CZ-5985	1629	0	58	MALE	G2	Stage II	T2	M0
TCGA-BP-4962	1785	0	58	MALE	G2	Stage II	T2	M0
TCGA-B2-3923	362	0	59	MALE	G2	Stage II	T2	M0
TCGA-CZ-5989	1599	0	60	MALE	G2	Stage II	T2	M0
TCGA-CZ-4861	446	1	63	MALE	G2	Stage II	T2	M0

TCGA-CZ-5453	25	0	67	MALE	G2	Stage II	T2	M0
TCGA-A3-3317	1491	0	67	MALE	G2	Stage II	T2	M0
TCGA-CZ-5452	1556	0	69	MALE	G2	Stage II	T2	M0
TCGA-CZ-5463	662	0	76	MALE	G2	Stage II	T2	M0
TCGA-AK-3447	1217	0	83	MALE	G2	Stage II	T2	M0
TCGA-AK-3431	1853	0	62	FEMALE	G3	Stage II	T2	M0
TCGA-CJ-6032	2548	0	63	FEMALE	G3	Stage II	T2	M0
TCGA-B0-4818	510	1	68	FEMALE	G3	Stage II	T2	M0
TCGA-CZ-5470	0	0	72	FEMALE	G3	Stage II	T2	M0
TCGA-BP-4960	2172	0	46	MALE	G3	Stage II	T2	M0
TCGA-BP-4173	1893	0	47	MALE	G3	Stage II	T2	M0
TCGA-AK-3456	1143	0	48	MALE	G3	Stage II	T2	M0
TCGA-AK-3451	1481	0	48	MALE	G3	Stage II	T2	M0
TCGA-BP-4174	1879	0	49	MALE	G3	Stage II	T2	M0
TCGA-B0-4816	1371	1	49	MALE	G3	Stage II	T2	M0
TCGA-A3-3316	1493	0	57	MALE	G3	Stage II	T2	M0
TCGA-CZ-5456	1558	0	57	MALE	G3	Stage II	T2	M0
TCGA-CJ-4912	1657	0	61	MALE	G3	Stage II	T2	M0
TCGA-A3-3357	2688	0	62	MALE	G3	Stage II	T2	M0
TCGA-CZ-5451	1668	0	74	MALE	G3	Stage II	T2	M0
TCGA-A3-3343	945	0	79	MALE	G3	Stage II	T2	M0
TCGA-BP-4342	2256	1	79	MALE	G3	Stage II	T2	M0
TCGA-CZ-4864	1315	1	86	MALE	G3	Stage II	T2	M0
TCGA-CZ-4858	1943	0	39	MALE	G4	Stage II	T2	M0
TCGA-BP-5200	1063	0	44	MALE	G4	Stage II	T2	M0
TCGA-BP-5199	1355	0	58	MALE	G4	Stage II	T2	M0
TCGA-B0-4822	1111	1	78	MALE	G4	Stage II	T2	M0
TCGA-CJ-4869	2554	0	49	MALE	G2	Stage III	T2	M0
TCGA-A3-3351	910	0	42	MALE	G2	Stage II	T2a	M0
TCGA-B8-5162	22	0	62	MALE	G2	Stage II	T2a	M0
TCGA-B2-4101	188	0	52	MALE	G3	Stage II	T2a	M0
TCGA-CJ-5675	2430	0	70	MALE	G3	Stage II	T2a	M0
TCGA-A3-3335	1886	0	41	MALE	G4	Stage II	T2a	M0
TCGA-CJ-4643	1793	0	67	FEMALE	G3	Stage II	T2b	M0
TCGA-CJ-4876	1955	0	57	MALE	G3	Stage II	T2b	M0
TCGA-A3-3372	735	0	64	MALE	G2	Stage III	T3	M0
TCGA-B8-4151	280	0	51	FEMALE	G2	Stage III	T3a	M0
TCGA-BP-4351	970	0	51	FEMALE	G2	Stage III	T3a	M0
TCGA-BP-4164	992	1	51	FEMALE	G2	Stage III	T3a	M0
TCGA-CJ-4870	1498	0	58	FEMALE	G2	Stage III	T3a	M0
TCGA-BP-4330	1888	0	60	FEMALE	G2	Stage III	T3a	M0
TCGA-B0-5113	359	0	69	FEMALE	G2	Stage III	T3a	M0
TCGA-B8-4620	226	0	70	FEMALE	G2	Stage III	T3a	M0
TCGA-CJ-4878	2186	0	71	FEMALE	G2	Stage III	T3a	M0

TCGA-B0-5075	637	1	77	FEMALE	G2	Stage III	T3a	M0
TCGA-BP-4332	1133	0	36	MALE	G2	Stage III	T3a	M0
TCGA-B0-5108	911	0	54	MALE	G2	Stage III	T3a	M0
TCGA-B0-4718	616	0	57	MALE	G2	Stage III	T3a	M0
TCGA-BP-4167	2718	0	59	MALE	G2	Stage III	T3a	M0
TCGA-CJ-5684	2231	0	61	MALE	G2	Stage III	T3a	M0
TCGA-CZ-5466	685	0	67	MALE	G2	Stage III	T3a	M0
TCGA-BP-4160	2881	0	67	MALE	G2	Stage III	T3a	M0
TCGA-BP-4341	1589	1	67	MALE	G2	Stage III	T3a	M0
TCGA-BP-5202	29	0	75	MALE	G2	Stage III	T3a	M0
TCGA-BP-4329	845	1	75	MALE	G2	Stage III	T3a	M0
TCGA-BP-4967	205	0	76	MALE	G2	Stage III	T3a	M0
TCGA-BP-5191	967	0	79	MALE	G2	Stage III	T3a	M0
TCGA-BP-4972	1502	0	43	FEMALE	G3	Stage III	T3a	M0
TCGA-BP-4163	2839	0	60	FEMALE	G3	Stage III	T3a	M0
TCGA-B0-5709	3117	0	62	FEMALE	G3	Stage III	T3a	M0
TCGA-B8-5163	4	0	63	FEMALE	G3	Stage III	T3a	M0
TCGA-CJ-4916	1373	0	69	FEMALE	G3	Stage III	T3a	M0
TCGA-CJ-4884	1759	0	72	FEMALE	G3	Stage III	T3a	M0
TCGA-B0-4710	96	0	75	FEMALE	G3	Stage III	T3a	M0
TCGA-BP-4981	1097	1	75	FEMALE	G3	Stage III	T3a	M0
TCGA-B0-5085	770	1	76	FEMALE	G3	Stage III	T3a	M0
TCGA-CJ-4897	1808	0	79	FEMALE	G3	Stage III	T3a	M0
TCGA-CJ-4873	1776	0	85	FEMALE	G3	Stage III	T3a	M0
TCGA-AK-3426	885	1	37	MALE	G3	Stage III	T3a	M0
TCGA-BP-4971	1487	0	40	MALE	G3	Stage III	T3a	M0
TCGA-CJ-4881	2014	0	41	MALE	G3	Stage III	T3a	M0
TCGA-CZ-5458	1558	0	43	MALE	G3	Stage III	T3a	M0
TCGA-BP-4973	1384	0	47	MALE	G3	Stage III	T3a	M0
TCGA-B0-4810	478	1	47	MALE	G3	Stage III	T3a	M0
TCGA-B0-4811	1417	1	48	MALE	G3	Stage III	T3a	M0
TCGA-CJ-4636	1924	0	51	MALE	G3	Stage III	T3a	M0
TCGA-B0-4849	69	1	51	MALE	G3	Stage III	T3a	M0
TCGA-BP-4334	645	1	56	MALE	G3	Stage III	T3a	M0
TCGA-BP-5183	1291	0	57	MALE	G3	Stage III	T3a	M0
TCGA-CJ-4882	1883	0	57	MALE	G3	Stage III	T3a	M0
TCGA-B0-4843	320	1	57	MALE	G3	Stage III	T3a	M0
TCGA-BP-4989	118	0	58	MALE	G3	Stage III	T3a	M0
TCGA-CJ-4894	841	1	58	MALE	G3	Stage III	T3a	M0
TCGA-B0-4696	866	1	58	MALE	G3	Stage III	T3a	M0
TCGA-CJ-4902	1520	0	61	MALE	G3	Stage III	T3a	M0
TCGA-BP-4343	1912	1	64	MALE	G3	Stage III	T3a	M0
TCGA-B8-5164	23	0	65	MALE	G3	Stage III	T3a	M0
TCGA-BP-4166	13	0	69	MALE	G3	Stage III	T3a	M0

TCGA-AK-3445	1280	0	69	MALE	G3	Stage III	T3a	M0
TCGA-B8-5550	434	0	71	MALE	G3	Stage III	T3a	M0
TCGA-B0-5694	480	1	71	MALE	G3	Stage III	T3a	M0
TCGA-B0-5100	1913	1	72	MALE	G3	Stage III	T3a	M0
TCGA-B8-4153	405	0	74	MALE	G3	Stage III	T3a	M0
TCGA-A3-3352	561	1	74	MALE	G3	Stage III	T3a	M0
TCGA-BP-4803	204	0	79	MALE	G3	Stage III	T3a	M0
TCGA-EU-5907	127	0	81	MALE	G3	Stage III	T3a	M0
TCGA-B0-5095	245	1	81	MALE	G3	Stage III	T3a	M0
TCGA-CJ-4913	1173	1	45	FEMALE	G4	Stage III	T3a	M0
TCGA-BP-4355	953	1	59	FEMALE	G4	Stage III	T3a	M0
TCGA-BP-4983	1413	0	67	FEMALE	G4	Stage III	T3a	M0
TCGA-B0-4693	77	1	72	FEMALE	G4	Stage III	T3a	M0
TCGA-B0-4842	1724	1	73	FEMALE	G4	Stage III	T3a	M0
TCGA-CZ-5467	73	1	86	FEMALE	G4	Stage III	T3a	M0
TCGA-CW-6097	571	1	32	MALE	G4	Stage III	T3a	M0
TCGA-CJ-4640	1998	0	49	MALE	G4	Stage III	T3a	M0
TCGA-B8-5158	293	0	56	MALE	G4	Stage III	T3a	M0
TCGA-BP-4761	182	0	57	MALE	G4	Stage III	T3a	M0
TCGA-B0-4706	65	1	61	MALE	G4	Stage III	T3a	M0
TCGA-CZ-5457	1547	0	62	MALE	G4	Stage III	T3a	M0
TCGA-B0-4707	600	1	63	MALE	G4	Stage III	T3a	M0
TCGA-BP-5010	878	1	63	MALE	G4	Stage III	T3a	M0
TCGA-B0-4815	1588	1	65	MALE	G4	Stage III	T3a	M0
TCGA-B0-5696	1727	0	69	MALE	G4	Stage III	T3a	M0
TCGA-BP-4985	952	1	72	MALE	G4	Stage III	T3a	M0
TCGA-B0-5097	665	0	59	FEMALE	G2	Stage III	T3b	M0
TCGA-CW-5587	2226	0	62	FEMALE	G2	Stage III	T3b	M0
TCGA-CZ-5465	1377	0	76	FEMALE	G2	Stage III	T3b	M0
TCGA-B0-4713	202	1	76	FEMALE	G2	Stage III	T3b	M0
TCGA-A3-3308	16	0	77	FEMALE	G2	Stage III	T3b	M0
TCGA-B0-5081	362	1	79	FEMALE	G2	Stage III	T3b	M0
TCGA-AK-3428	2223	0	62	MALE	G2	Stage III	T3b	M0
TCGA-B4-5832	155	0	65	MALE	G2	Stage III	T3b	M0
TCGA-BP-4347	1367	0	74	MALE	G2	Stage III	T3b	M0
TCGA-CZ-4863	1691	0	51	FEMALE	G3	Stage III	T3b	M0
TCGA-B0-5692	1487	0	66	FEMALE	G3	Stage III	T3b	M0
TCGA-B0-4821	1230	1	68	FEMALE	G3	Stage III	T3b	M0
TCGA-AK-3455	683	1	71	FEMALE	G3	Stage III	T3b	M0
TCGA-B0-5713	1865	0	75	FEMALE	G3	Stage III	T3b	M0
TCGA-B0-5099	485	1	88	FEMALE	G3	Stage III	T3b	M0
TCGA-BP-4797	1107	0	34	MALE	G3	Stage III	T3b	M0
TCGA-CJ-4901	1450	0	47	MALE	G3	Stage III	T3b	M0
TCGA-CJ-5676	2575	0	47	MALE	G3	Stage III	T3b	M0

TCGA-B0-5711	2931	0	50	MALE	G3	Stage III	T3b	M0
TCGA-B0-5116	657	0	52	MALE	G3	Stage III	T3b	M0
TCGA-B0-4848	883	1	54	MALE	G3	Stage III	T3b	M0
TCGA-BP-4346	1493	1	57	MALE	G3	Stage III	T3b	M0
TCGA-CJ-4907	1499	0	58	MALE	G3	Stage III	T3b	M0
TCGA-AK-3430	480	1	61	MALE	G3	Stage III	T3b	M0
TCGA-BP-4345	1516	0	62	MALE	G3	Stage III	T3b	M0
TCGA-CZ-5459	1515	0	63	MALE	G3	Stage III	T3b	M0
TCGA-A3-3307	1436	0	66	MALE	G3	Stage III	T3b	M0
TCGA-B0-4813	18	1	68	MALE	G3	Stage III	T3b	M0
TCGA-BP-4799	1133	1	70	MALE	G3	Stage III	T3b	M0
TCGA-BP-5198	603	0	72	MALE	G3	Stage III	T3b	M0
TCGA-CW-5584	164	1	74	MALE	G3	Stage III	T3b	M0
TCGA-B0-5400	1132	0	59	FEMALE	G4	Stage III	T3b	M0
TCGA-BP-4337	2	1	76	FEMALE	G4	Stage III	T3b	M0
TCGA-B0-4827	885	1	77	FEMALE	G4	Stage III	T3b	M0
TCGA-B0-5701	1732	0	65	MALE	G4	Stage III	T3b	M0
TCGA-B0-5109	587	1	69	MALE	G4	Stage III	T3b	M0
TCGA-B0-4694	106	1	72	MALE	G4	Stage III	T3b	M0
TCGA-CJ-5679	679	1	73	MALE	G4	Stage III	T3b	M0
TCGA-B0-4817	1019	1	81	MALE	G3	Stage III	T3c	M0
TCGA-CJ-4891	819	1	57	FEMALE	G4	Stage III	T3c	M0
TCGA-BP-4770	329	1	73	FEMALE	G4	Stage IV	T4	M0
TCGA-B0-5402	449	0	64	MALE	G4	Stage IV	T4	M0
TCGA-B0-4698	42	1	75	MALE	G4	Stage IV	T4	M0
TCGA-B0-5092	459	1	53	FEMALE	G3	Stage IV	T1a	M1
TCGA-CZ-5462	311	1	83	MALE	G3	Stage IV	T1b	M1
TCGA-G6-A5PC	242	1	54	FEMALE	G4	Stage IV	T1b	M1
TCGA-CZ-5461	330	1	52	MALE	G4	Stage IV	T1b	M1
TCGA-AK-3436	2044	0	40	MALE	G2	Stage IV	T2	M1
TCGA-CZ-5454	722	1	63	MALE	G2	Stage IV	T2	M1
TCGA-B0-5712	2722	0	68	FEMALE	G3	Stage IV	T2	M1
TCGA-B0-5115	797	0	43	MALE	G3	Stage IV	T2	M1
TCGA-B0-4691	139	1	55	MALE	G3	Stage IV	T2	M1
TCGA-B0-4841	204	1	63	MALE	G3	Stage IV	T2	M1
TCGA-B0-4828	307	1	79	MALE	G3	Stage IV	T2	M1
TCGA-B0-5107	927	1	65	FEMALE	G4	Stage IV	T2	M1
TCGA-CJ-5678	574	1	62	MALE	G3	Stage IV	T2b	M1
TCGA-CJ-4637	2227	1	52	FEMALE	G4	Stage IV	T2b	M1
TCGA-B4-5377	365	0	68	FEMALE	G3	Stage IV	T3	M1
TCGA-B2-5639	417	0	46	MALE	G3	Stage IV	T3	M1
TCGA-B0-4846	1200	1	52	MALE	G2	Stage IV	T3a	M1
TCGA-CW-5591	2271	0	56	MALE	G2	Stage IV	T3a	M1
TCGA-B0-4845	1986	1	70	MALE	G2	Stage IV	T3a	M1

TCGA-CJ-5681	552	1	44	FEMALE	G3	Stage IV	T3a	M1
TCGA-CJ-4644	336	1	48	FEMALE	G3	Stage IV	T3a	M1
TCGA-CJ-4904	1792	0	60	FEMALE	G3	Stage IV	T3a	M1
TCGA-BP-4335	475	1	65	FEMALE	G3	Stage IV	T3a	M1
TCGA-B0-4701	238	1	66	FEMALE	G3	Stage IV	T3a	M1
TCGA-B8-4143	709	1	66	FEMALE	G3	Stage IV	T3a	M1
TCGA-CW-5580	1964	1	73	FEMALE	G3	Stage IV	T3a	M1
TCGA-B0-5084	222	1	33	MALE	G3	Stage IV	T3a	M1
TCGA-CJ-4868	646	1	42	MALE	G3	Stage IV	T3a	M1
TCGA-CJ-4887	932	1	48	MALE	G3	Stage IV	T3a	M1
TCGA-CW-5590	1075	1	51	MALE	G3	Stage IV	T3a	M1
TCGA-CZ-4857	1432	1	56	MALE	G3	Stage IV	T3a	M1
TCGA-B8-4622	181	0	57	MALE	G3	Stage IV	T3a	M1
TCGA-B0-4844	313	1	60	MALE	G3	Stage IV	T3a	M1
TCGA-B0-4847	793	1	60	MALE	G3	Stage IV	T3a	M1
TCGA-B0-5080	342	1	63	MALE	G3	Stage IV	T3a	M1
TCGA-CJ-4885	2125	0	64	MALE	G3	Stage IV	T3a	M1
TCGA-CJ-4875	2353	0	67	MALE	G3	Stage IV	T3a	M1
TCGA-B0-4712	1337	1	76	MALE	G3	Stage IV	T3a	M1
TCGA-CJ-4638	431	1	46	FEMALE	G4	Stage IV	T3a	M1
TCGA-CJ-6033	224	1	54	FEMALE	G4	Stage IV	T3a	M1
TCGA-CJ-5677	782	1	54	FEMALE	G4	Stage IV	T3a	M1
TCGA-CJ-4641	1661	1	55	FEMALE	G4	Stage IV	T3a	M1
TCGA-BP-4787	480	1	59	FEMALE	G4	Stage IV	T3a	M1
TCGA-CJ-4923	572	1	63	FEMALE	G4	Stage IV	T3a	M1
TCGA-CJ-5680	768	1	65	FEMALE	G4	Stage IV	T3a	M1
TCGA-B0-4703	182	1	51	MALE	G4	Stage IV	T3a	M1
TCGA-BP-4974	211	1	58	MALE	G4	Stage IV	T3a	M1
TCGA-CJ-6028	1625	1	58	MALE	G4	Stage IV	T3a	M1
TCGA-CJ-4888	1567	1	59	MALE	G4	Stage IV	T3a	M1
TCGA-CJ-5682	1883	0	60	MALE	G4	Stage IV	T3a	M1
TCGA-CW-6087	41	1	61	MALE	G4	Stage IV	T3a	M1
TCGA-BP-4771	162	1	62	MALE	G4	Stage IV	T3a	M1
TCGA-CJ-4895	1200	1	62	MALE	G4	Stage IV	T3a	M1
TCGA-CJ-4871	2423	0	63	MALE	G4	Stage IV	T3a	M1
TCGA-CJ-4918	93	1	64	MALE	G4	Stage IV	T3a	M1
TCGA-BP-5178	1912	1	71	MALE	G4	Stage IV	T3a	M1
TCGA-CJ-4890	2085	0	72	MALE	G4	Stage IV	T3a	M1
TCGA-CW-5585	2609	0	51	MALE	G2	Stage IV	T3b	M1
TCGA-CZ-5460	1430	0	55	MALE	G2	Stage IV	T3b	M1
TCGA-CZ-5987	445	1	60	MALE	G2	Stage IV	T3b	M1
TCGA-B0-5094	333	1	62	MALE	G2	Stage IV	T3b	M1
TCGA-CZ-5464	1492	0	69	MALE	G2	Stage IV	T3b	M1
TCGA-B0-4836	1238	1	61	MALE	G3	Stage IV	T3b	M1

TCGA-B0-4714	99	1	81	MALE	G3	Stage IV	T3b	M1
TCGA-B0-4697	578	1	46	FEMALE	G4	Stage IV	T3b	M1
TCGA-B0-4819	183	1	60	FEMALE	G4	Stage IV	T3b	M1
TCGA-BP-4352	344	1	74	FEMALE	G4	Stage IV	T3b	M1
TCGA-BP-5201	951	0	63	MALE	G4	Stage IV	T3b	M1
TCGA-CZ-5455	561	1	63	MALE	G4	Stage IV	T3b	M1
TCGA-CZ-5468	59	1	84	MALE	G4	Stage IV	T3b	M1
TCGA-B0-4814	168	1	58	MALE	G3	Stage IV	T4	M1
TCGA-B0-4690	43	1	65	MALE	G3	Stage IV	T4	M1
TCGA-CJ-4900	1714	1	69	FEMALE	G4	Stage IV	T4	M1
TCGA-BP-4354	1034	1	40	MALE	G4	Stage IV	T4	M1
TCGA-B0-4688	101	1	46	MALE	G4	Stage IV	T4	M1
TCGA-CZ-4860	206	1	60	MALE	G4	Stage IV	T4	M1
TCGA-B0-4700	1980	1	60	MALE	G4	Stage IV	T4	M1
TCGA-B0-4699	110	1	74	MALE	G4	Stage IV	T4	M1
TCGA-T7-A92I	356	0	47	FEMALE	G1	Stage I	T1a	MX
