# Supplementary figures and images for: Effects and Prognostic Values of Circadian Genes CSNK1E/GNA11/KLF9/THRAP3 in Kidney Renal Clear Cell Carcinoma via a Comprehensive Analysis
Source: Bioengineering (Basel). 2022 Jul 11;9(7):306. doi: 10.3390/bioengineering9070306 (PMC9311602; doi:10.3390/bioengineering9070306)

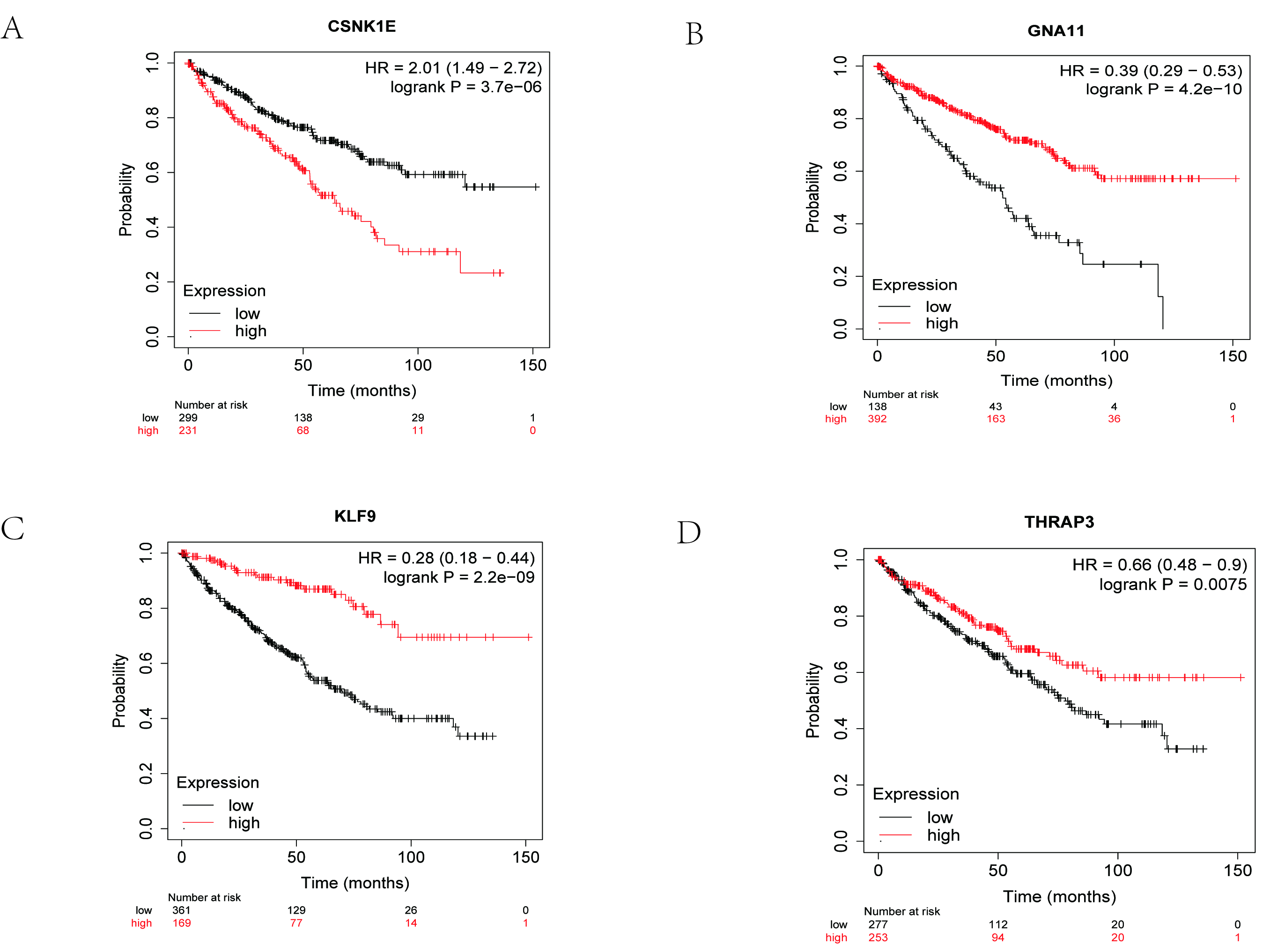

Supplement: Supplementary file 1 [file bioengineering-09-00306-s001.zip › Supplementary Figure.S3.tif]

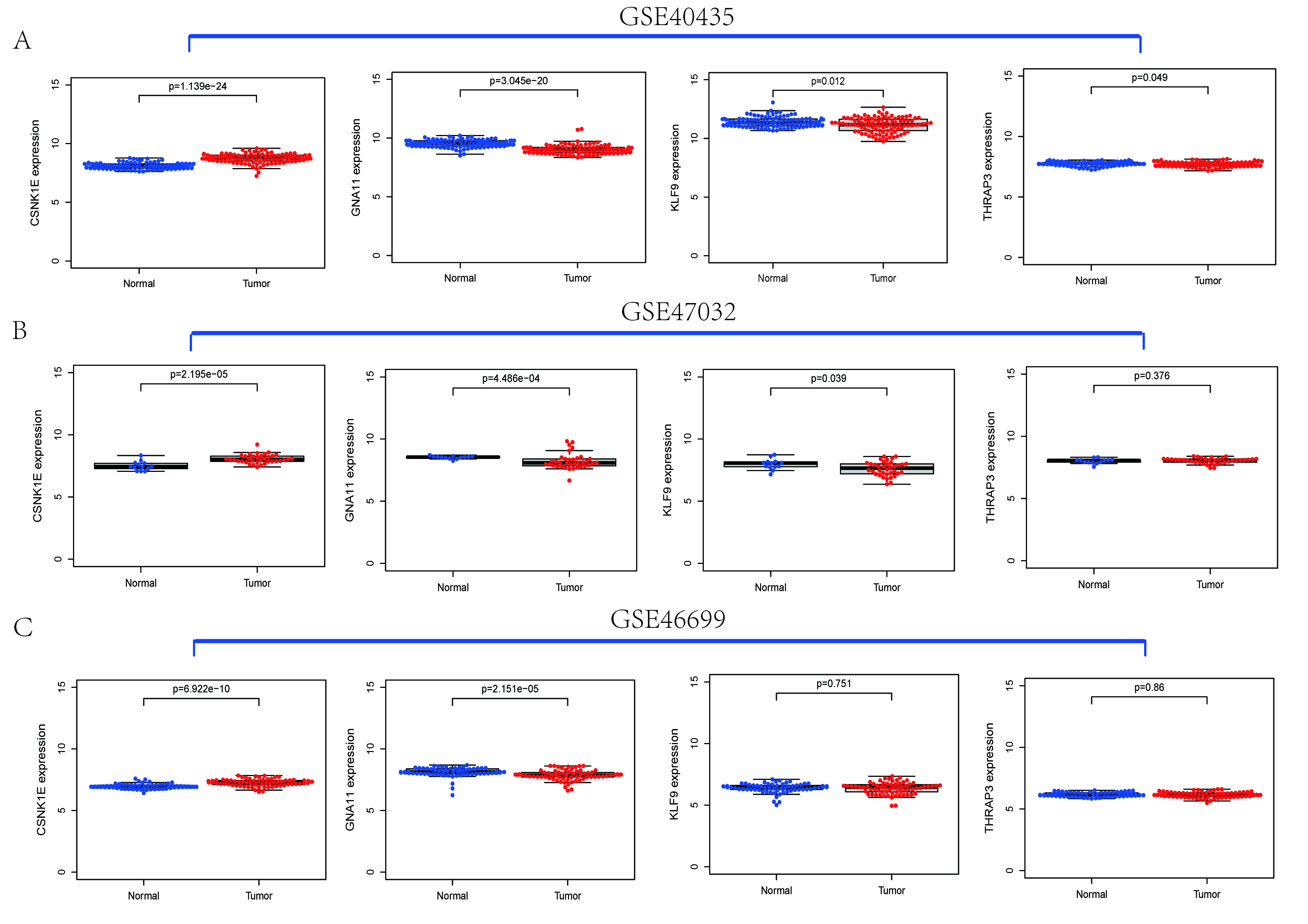

Supplement: Supplementary file 1 [file bioengineering-09-00306-s001.zip › Supplementary Figure.S5.tif]

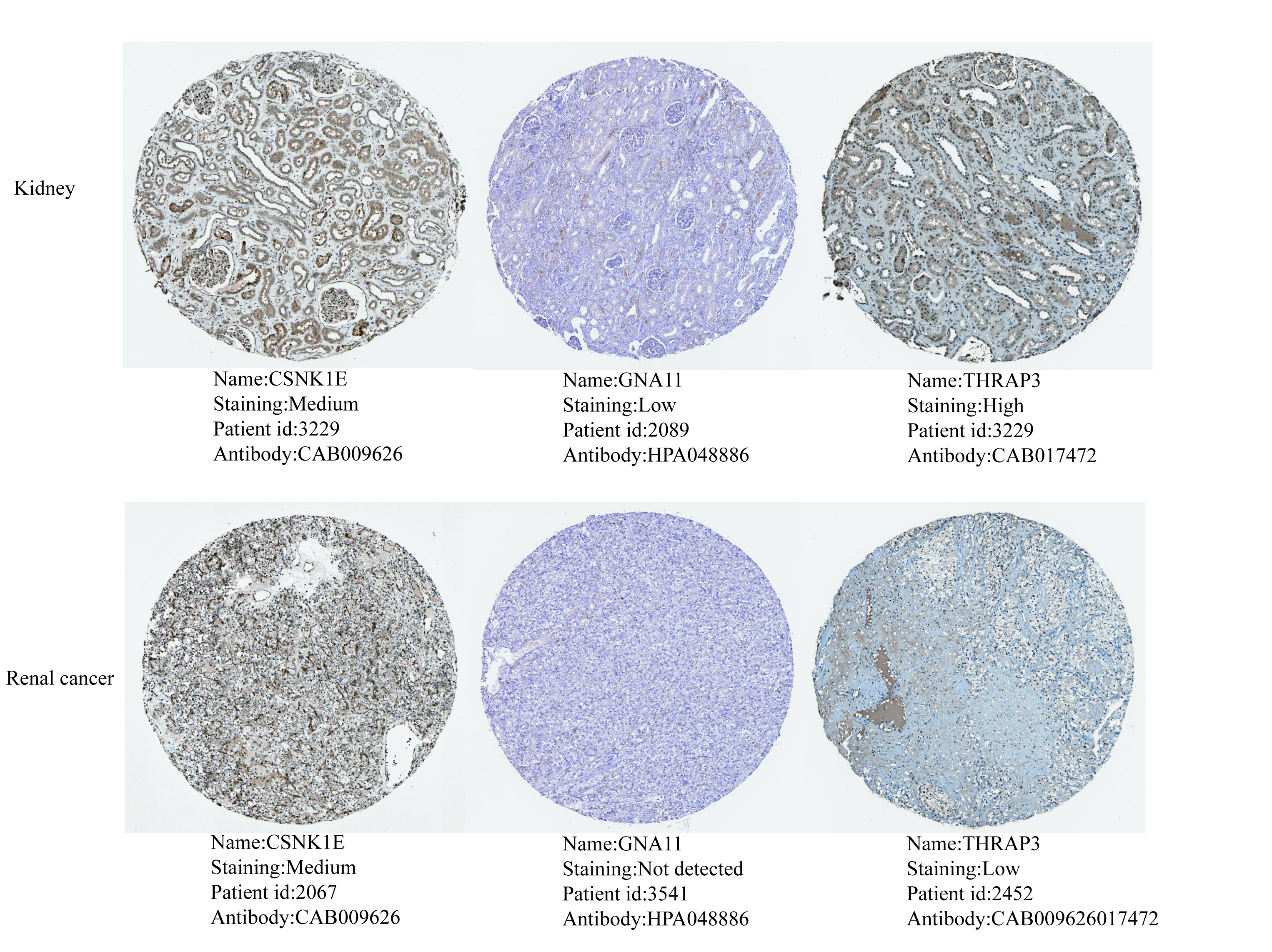

Supplement: Supplementary file 1 [file bioengineering-09-00306-s001.zip › Supplementary Figure.S6.tif]

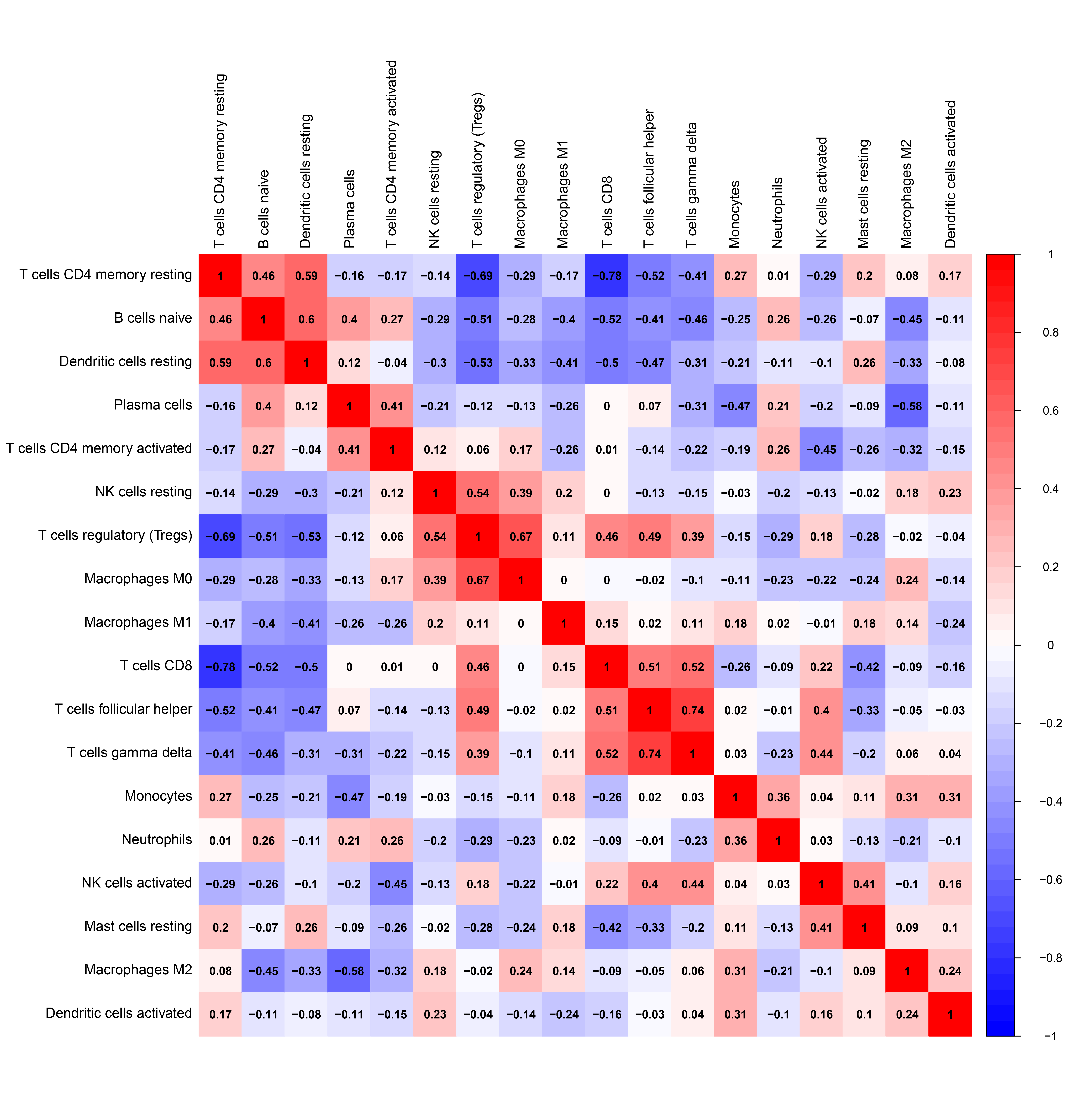

Supplement: Supplementary file 1 [file bioengineering-09-00306-s001.zip › Supplementary Figure.S8.tif]

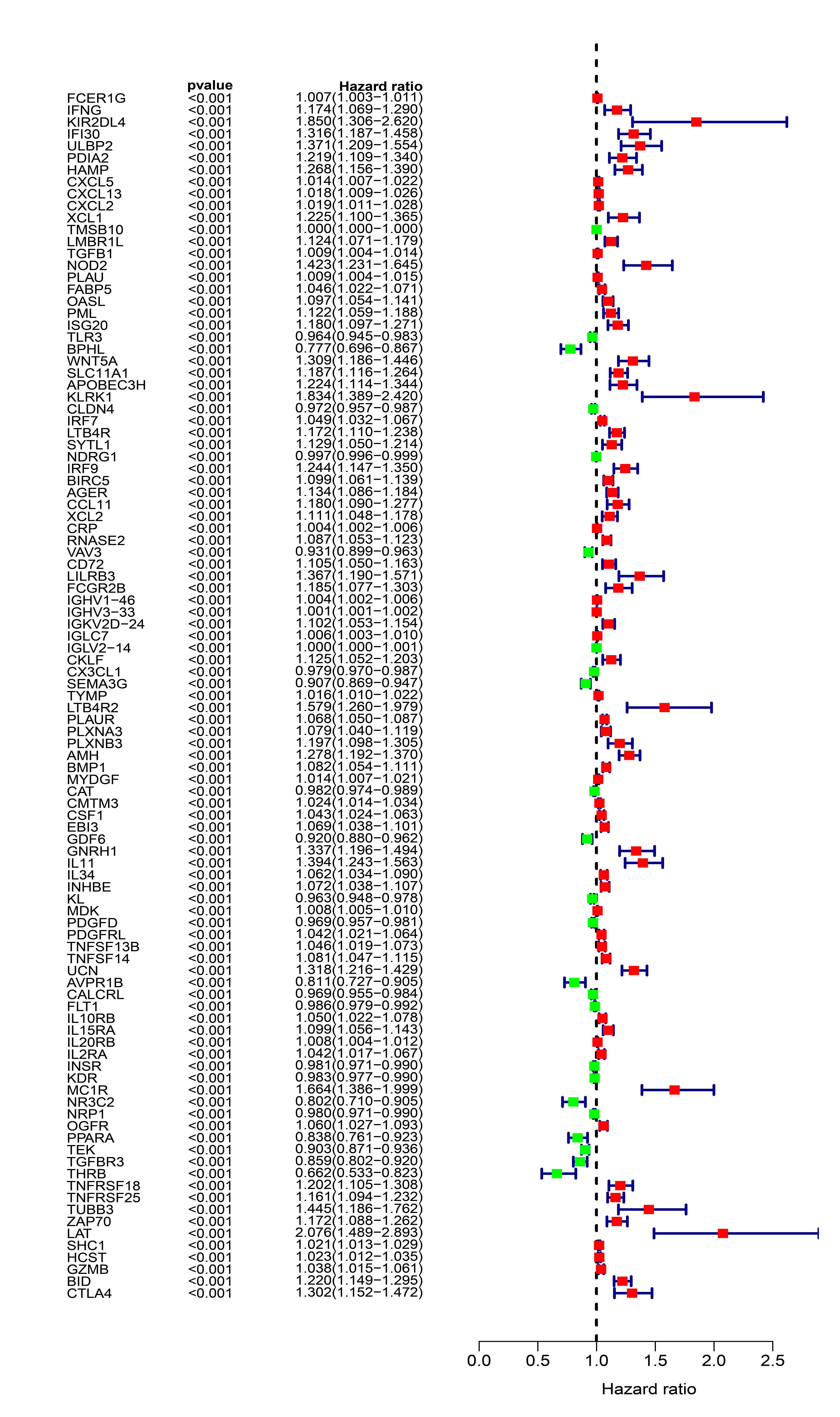

Supplement: Supplementary file 1 [file bioengineering-09-00306-s001.zip › Supplementary Figure.S9.tif]
